# Mobile-Based Digital Rehabilitation Program for Patients After Anterior Cervical Discectomy and Fusion: Prospective Cohort Study

**DOI:** 10.2196/60717

**Published:** 2025-12-01

**Authors:** Sen Liu, Xin Chen, Di Liu, Crystal Ye Lin, Yaping Chen, Siyi Cai

**Affiliations:** 1 Department of Orthopeadics, Peking Union Medical College Hospital, Chinese Academy of Medical Sciences (PUMCH-CAMS), No. 1 Shuaifuyuan, Dongcheng District, Beijing, 100730, China, 86 13810003291; 2 Spinal Surgery Department, Jishuitan Hospital Affiliated to Capital Medical University, Beijing, China; 3 Medical Innovation Department, Jiakang Zhongzhi Technology Company, Beijing, China; 4 Pritzker School of Medicine, University of Chicago, Chicago, IL, United States

**Keywords:** anterior cervical discectomy and fusion, digital rehabilitation, computer vision, remote rehabilitation, wearable sensors, postoperative rehabilitation

## Abstract

**Background:**

Anterior cervical discectomy and fusion (ACDF) is a common treatment for degenerative cervical spine disease, yet its frequent postoperative follow-up places substantial demands on both patients and health care systems. A digital program integrating computer-vision–guided exercise, wearable posture monitoring, and cognitive behavioral therapy (CBT) could provide remote monitoring and rehabilitation to alleviate this burden.

**Objective:**

This study aims to evaluate the clinical effectiveness and compliance of a 12-week digital rehabilitation program after ACDF compared with conventional in-person therapy.

**Methods:**

In this prospective cohort study, 336 postoperative patients self-selected either a 12-week mobile-based program incorporating computer-vision–guided exercises, wearable posture sensors, and cognitive behavioral therapy (n=270), or in-person rehabilitation group (IRG, n=66) involving weekly therapist-supervised sessions and paper-based home exercises. Digital users were stratified into a digital rehabilitation completion group (DCG, n=192) and a digital rehabilitation noncompletion group (DNG, n=78). All participants were recruited at a single tertiary hospital and returned to the clinic for outcome assessments at 0, 12, and 24 weeks postoperatively. Outcomes—primarily pain (visual analog scale [VAS]) and disability (neck disability index [NDI]), as well as 36-item short form survey mental component summary (SF-36 MCS), 3-plane cervical range of motion (ROM), muscle endurance, and patient satisfaction—were recorded at 0, 12, and 24 weeks postoperatively. All statistical analyses were performed using SPSS (version 29.0; IBM Corporation). Results were reported as means, SDs, and 95% CIs.

**Results:**

Both the DCG (n=192, who completed all digital sessions) and IRG (who completed 12 weeks of weekly in-person sessions and home exercises) demonstrated significant improvements in pain and disability at weeks 12 and 24, with no significant differences between groups (*P*>.05). At Week 12, VAS decreased by −2.5 (95% CI −3.0 to −2.0) in the DCG and −2.8 (−3.7 to −1.9) in the IRG; NDI decreased by −6.8 (−10.3 to −3.3) and −8.1 (−14.3 to −1.9), respectively. At Week 24, VAS and NDI reductions reached −4.0 (−4.5 to −3.5) and −13.3 (−17.4 to −9.2) in the DCG, and −4.1 (−5.0 to −3.2) and −14.2 (−21.3 to −7.1) in the IRG. In contrast, the DNG showed minimal improvements: VAS changes were −0.8 (−1.6 to 0.0) at week 12 and −1.3 (−2.1 to −0.5) at week 24; NDI changes were −2.2 (−8.2 to 3.8) and −6.4 (−13.0 to 0.2), respectively (*P*<.05 compared to DCG and IRG).

**Conclusions:**

The digital rehab program led to comparable improvements in pain, function, and mental health as conventional in-person rehab. Higher adherence was linked to better outcomes, supporting digital rehab as an effective, patient-centered approach after ACDF.

## Introduction

### Background

With the rapid global aging of the population, degenerative cervical spine disease has become a major public health challenge affecting older adults. Approximately 85% of individuals above 60 years old show radiographic evidence of cervical degeneration, and the number of people living with neck pain reached 203 million—an increase of 77.3% since 1990—placing neck pain among the leading contributors to musculoskeletal disability [1]. Also, the global age-standardized prevalence of neck pain is 27 per 1000 in 2019, causing substantial health care expenditure and productivity loss [2].

For degenerative cervical spine disease accompanied by spinal cord or nerve root compression, anterior cervical discectomy and fusion (ACDF) remains the surgical gold standard [3]. However, shorter inpatient stays indicate that most functional recovery occurs after discharge, and traditional outpatient physiotherapy often lacks continuous monitoring, real-time feedback, and psychological support [4]. It is further revealed that postoperative patients and their caregivers widely seek support to close these postdischarge guidance gaps [4].

Digital rehabilitation has the potential to address these shortcomings and provide remote, real-time, and individualized exercise guidance. In previous studies involving chronic low back pain, AI-assisted tele-rehabilitation significantly reduced pain and improved function within 4‐8 weeks [5,6], while systematic reviews confirmed that digital health interventions lessen neck and back pain and enhance quality of life [7,8]. Other studies further indicate that spine-surgery patients using digital platforms experience lower rates of postoperative emergency visits and readmissions, highlighting their potential in postoperative care [9].

However, evidence for digital rehabilitation specifically designed for ACDF postoperative patients remains limited [9]. Thus, this study evaluates the effectiveness and safety of an AI-driven digital rehabilitation program in promoting functional recovery and improving compliance following ACDF, thereby providing evidence-based postoperative digital rehabilitation strategies.

### Objective

This study aims to evaluate the clinical efficacy of a digital rehabilitation program that combines computer-vision–guided exercise coaching, real-time inertial-sensor monitoring, and cognitive behavioral interventions for patients recovering from ACDF. By systematically comparing neck-pain intensity, quality of life, and functional recovery before and after the intervention, we will assess the program’s effectiveness in relieving symptoms, enhancing quality of life, and promoting functional recovery.

## Methods

### Study Design

This single-centered, prospective cohort study was carried out at Peking Union Medical College Hospital to compare a 12-week digital rehabilitation program with standard in-person physiotherapy after ACDF [10-15]. A total of 336 eligible patients were enrolled.

Postoperatively, participants selected their rehabilitation modality based on personal preference and logistical convenience, forming the digital rehabilitation group and the in-person rehabilitation group (IRG). Within the digital rehabilitation group, compliance criteria further divided patients into a digital rehabilitation completion group (DCG) and a digital rehabilitation noncompletion group (DNG).

All groups followed an identical evidence-based rehabilitation protocol. Digital groups (DCG and DNG) used the healbone rehabilitation system (HRS), a remote rehabilitation platform developed by Jiakangzhongzhi Technology Co, paired with inertial measurement unit (IMU) wearables, completing three 30-minute sessions per week. IRG attended one 45-minute, in-person session with a physical therapist each week and performed the remaining exercises independently at home using a printed manual. Pain, functional status, health-related quality of life, and cervical range of motion were assessed at baseline (Week 0), Week 12, and Week 24, whereas patient satisfaction was measured only at Week 24.

### Inclusion and Exclusion Criteria

All participants were recruited at Peking Union Medical College Hospital. Two licensed physicians enrolled postoperative ACDF candidates according to the inclusion and exclusion criteria. Each participant received detailed information about the study’s objectives, procedures, and potential risks. Enrollment proceeded only after written informed consent was obtained. The inclusion and exclusion criteria were as follows:

Textbox 1. **Inclusion and exclusion criteria for participant selection.**

**Inclusion criteria**:

1. Less than 70 years old.
2. Signed informed consent.
3. Diagnosis of cervical pathology Grade III or IV.
4. Experiencing cervical spine disease for longer than 2 months.
5. ≤3 segments requiring cervical fusion.

**Exclusion criteria**

1. Severe spinal canal stenosis.
2. Presence of diseases preventing exercise.
3. History of cervical or other spinal surgeries.
4. History of spinal infection or tumors.

### Blinding

Because of the distinct modes of intervention, conventional double‐blinding was not feasible. To minimize bias, we implemented several safeguards. Follow-up assessments were conducted by clinicians blinded to group allocation. Group identifiers were replaced with coded labels during data analysis, and data analysis was completed without knowledge of group allocation.

### Intervention

The digital and in-person groups followed an identical rehabilitation protocol, designed by licensed physicians and physical therapists with reference to clinical guidelines. The program included cervical range-of-motion exercises, cervical-muscle strengthening exercises, scapular-stability exercises, and functional exercises. Full details are provided in Multimedia Appendix 1. All patients underwent follow-up assessments at baseline, Week 12, and Week 24.

#### Digital Rehabilitation Group

**Figure 1.**
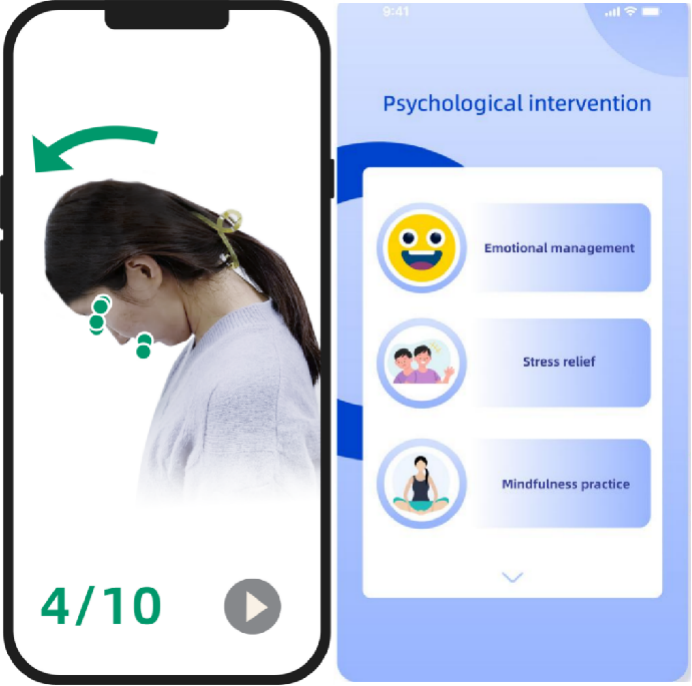
Computer vision–guided exercise and cognitive behavioral therapy modules. During each session, the front-facing cellphone camera works with a computer-vision-based software application to capture head-and-neck angles in real time for guided exercises. The cognitive behavioral therapy (CBT) module allows patients to access lessons and complete interactive homework assignments.

Participants used a mobile rehabilitation app paired with wearable IMU sensors for training and monitoring. They were instructed to complete 3 30-minute sessions per week for 12 weeks, totaling 36 sessions according to clinical protocols [16-18]. The app comprises three modules: (1) computer-vision exercise coaching, (2) cognitive behavioral therapy (CBT), and (3) posture monitoring. Figures1  2 depict the implementation of these modules.

**Figure 2.**
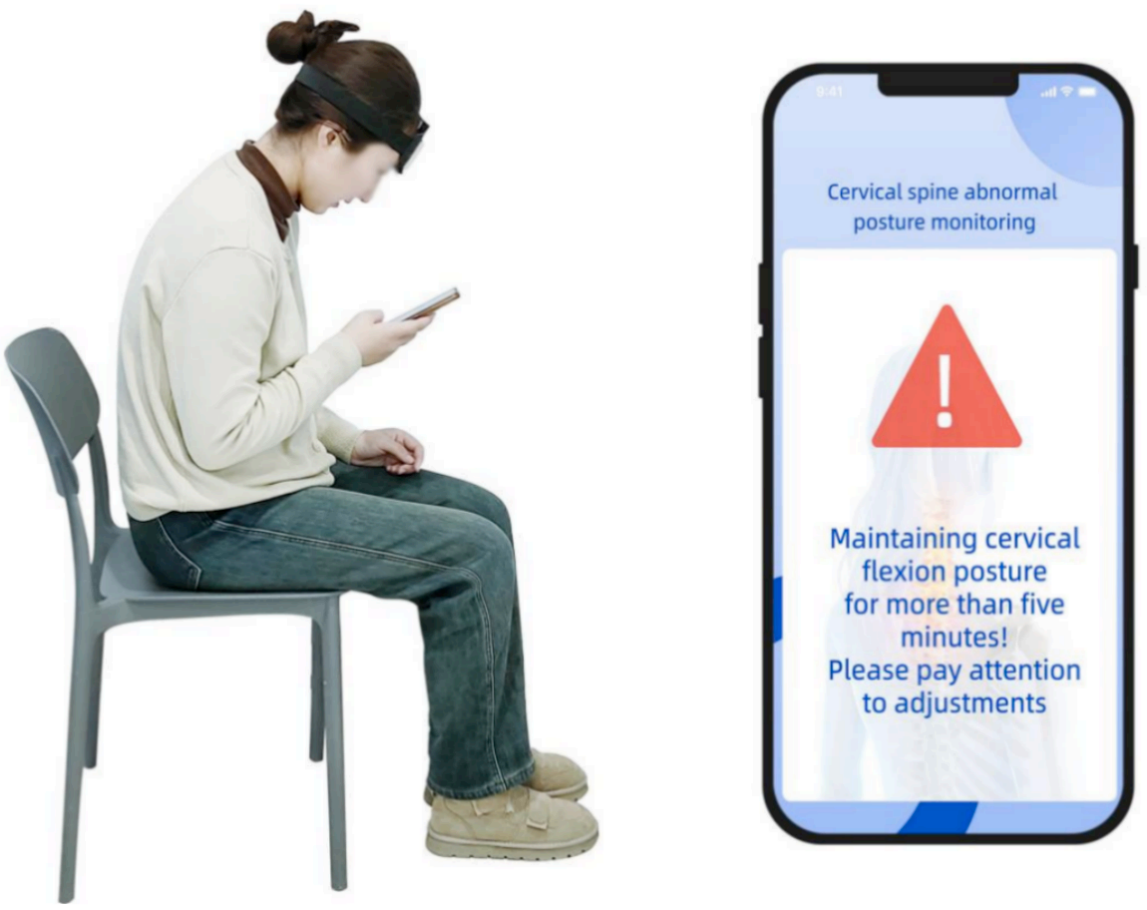
Patient monitoring module and software module. The posture-monitoring module uses a 9-axis inertial measurement unit (IMU) paired with a notification alert system. Patients wear the IMU during daily activities; when abnormal cervical posture is detected, the system sends a warning message, encouraging proper postoperative posture.

#### In-Person Rehabilitation Group

Participants attended one 45-minute, therapist-supervised session per week and received a printed home-exercise booklet identical to the digital protocol for self-directed practice in between visits. Two licensed physical therapists delivering the sessions completed 3 training workshops (≥1 hour each) before the beginning of this study to ensure protocol fidelity.

### Computer-Vision Exercise Coaching

Before discharge from the hospital, participants installed the training app on their smartphones and logged in with their registered accounts. Patients then followed the software app, which provided tutorials on how to complete each exercise. The cell phone’s front-facing camera captured cervical angles in real time, and the software provided verbal and auditory feedback to patients when they were completing their exercises in real time [19-22]. Each session lasted about 30 minutes, 3 times per week, for a total of 36 sessions over 12 weeks according to clinical guidelines [23-27].

### Cognitive Behavioral Therapy (CBT)

The digital rehabilitation app delivers eight micro-lessons designed to reduce postoperative chronic pain and psychological stress. Each lesson lasts 15‐20 minutes and is completed once per week during the first 8 weeks of training. Licensed therapists created the curriculum with reference to clinical protocols. The lessons progress through the following topics:

- Pain education: explaining that pain is not always proportional to tissue damage
- Cognitive restructuring: recognizing and correcting negative thought patterns
- Daily routine planning: helping patients establish balanced activity and rest schedules
- Sleep management: addressing postoperative insomnia or poor sleep quality
- Stress regulation: mitigating anxiety, fear, and feelings of helplessness
- Goal maintenance: reinforcing intrinsic motivation for long-term recovery.

After completing each lesson, patients complete an interactive homework assignment and write an electronic diary. Licensed therapists review submissions online each week and provide feedback within 24 hours, thereby fostering patients’ self-management skills.

### Posture Monitoring

Patients in the digital rehabilitation group were instructed to wear a 9-axis IMU on the forehead for real-time tracking of cervical flexion and rotation. The device had to be worn for more than 4 hours per day during waking activities. If forward flexion exceeded or axial rotation surpassed a preset range, the app sent an alert. Each day, the software generated a posture-monitoring report and uploaded it to the cloud for therapist review. If no data were received for 48 hours, the system issued an automatic reminder to the patient, followed by a therapist phone check-in to improve engagement [28-31].

### Completion Criteria

Within the digital rehabilitation arm, patients were classified as either the DCG or the DNG according to the following criteria:

Exercise compliance: all 36 scheduled sessions had to be completed. Each session required every prescribed exercise to be completed.

CBT compliance: all 8 CBT micro-lessons had to be viewed, with the associated homework submitted.

Posture-monitoring compliance: the IMU had to be worn for ≥4 hours per day, on ≥70% of the total days for the 12-week digital program [32-34].

Every DCG patient completed the full set of 36 sessions and met the CBT and posture-monitoring requirements. By contrast, most DNG participants discontinued the program early and did not meet completion criteria.

### Outcome Measures

#### Primary Outcomes

Primary outcomes included pain intensity, assessed with a visual analog scale (VAS), and neck-specific disability, assessed with the neck disability index (NDI). These measures are well validated in cervical spine disease populations [35]. We measured both outcomes at 0, 12, and 24 weeks postoperatively to capture the longitudinal course of pain relief and functional recovery.

#### Secondary Outcomes

##### Psychological Health

Psychological health was assessed with the mental component summary (MCS) of the 36-item short form survey (SF-36) [36]. Higher MCS values indicate better mental well-being. Participants complete the SF-36 at 0, 12, and 24 weeks after surgery, allowing us to track the trajectory of postoperative psychological adjustment alongside physical recovery.

##### Objective Kinematics

Cervical mobility was quantified as active range of motion (ROM) in 3 anatomical planes—flexion, extension, lateral bending, and axial rotation—using a handheld digital inclinometer. After a standardized warm-up, each participant performs 3 maximal pain-free movements per plane; the mean of the 3 trials is recorded at 0, 12, and 24 weeks to document changes in kinematics over time.

##### Muscle Endurance

Cervical flexor and extensor endurance was measured with standardized supine and prone hold tests as described by Harris et al [37]. For the flexor assessment, the participant was in a supine position, lifted their heads upwards, and maintained this posture until they felt fatigued. The duration of the test was measured in seconds. For the extensor assessment, positioned in a prone posture, participants suspended their necks in the air with a weight attached (2 kg for males, 1 kg for females) and maintained the neck parallel to the ground until they felt fatigued. The assessments were performed 3 times, and the mean of the 3 trials is recorded at 0, 12, and 24 weeks, providing an objective measure of muscular performance during rehabilitation.

##### Patient Experience

Overall patient experience was evaluated once, at 24 weeks, using the Chinese Patient Satisfaction Questionnaire–Revised (PSQ-R) [38]. Each item is rated on a 5-point Likert scale, with higher scores indicating greater satisfaction. Administering the PSQ-R after the full intervention period captures patients’ comprehensive impressions of care quality and rehabilitation effectiveness.

### Statistical Analysis

All statistical analyses were performed using SPSS software (version 29.0; IBM Corp). Descriptive statistics for baseline characteristics were reported as mean (SD) for continuous variables or number (%) for categorical variables. Between-group comparisons for continuous variables were conducted using independent-sample 2-tailed *t* tests under the assumption of normality. Categorical variables were compared using *χ*^2^ tests. Primary outcomes (VAS and NDI) and secondary outcomes (SF-36 MCS, cervical ROM, muscle endurance) were assessed at baseline (0 week), 12 weeks, and 24 weeks postoperatively. Results were presented as means, SDs, and 95% CIs. Pairwise group comparisons between the DCG, DNG, and IRG were conducted at each time point using independent-sample 2-tailed *t* tests. A *P* value less than .05 was considered statistically significant.

### Ethical Considerations

This study was supported by CAMS Innovation Fund for Medical Sciences (CIFMS; Grant No. 2023-I2M-C&T-B-002) and the National High-Level Hospital Clinical Research Funding (Grant No. 2022-PUMCH-A-123). The protocol adhered to the principles of the Declaration of Helsinki and was reviewed and approved by the Ethics Committee of Peking Union Medical College Hospital, Chinese Academy of Medical Sciences (approval No. I-25PJ0581). Written informed consent was obtained from all participants prior to enrollment. All personal data were coded and deidentified to ensure participant privacy and data security. No financial or other compensation was offered to participants.

## Results

### Participant Characteristics

**Figure 3.**
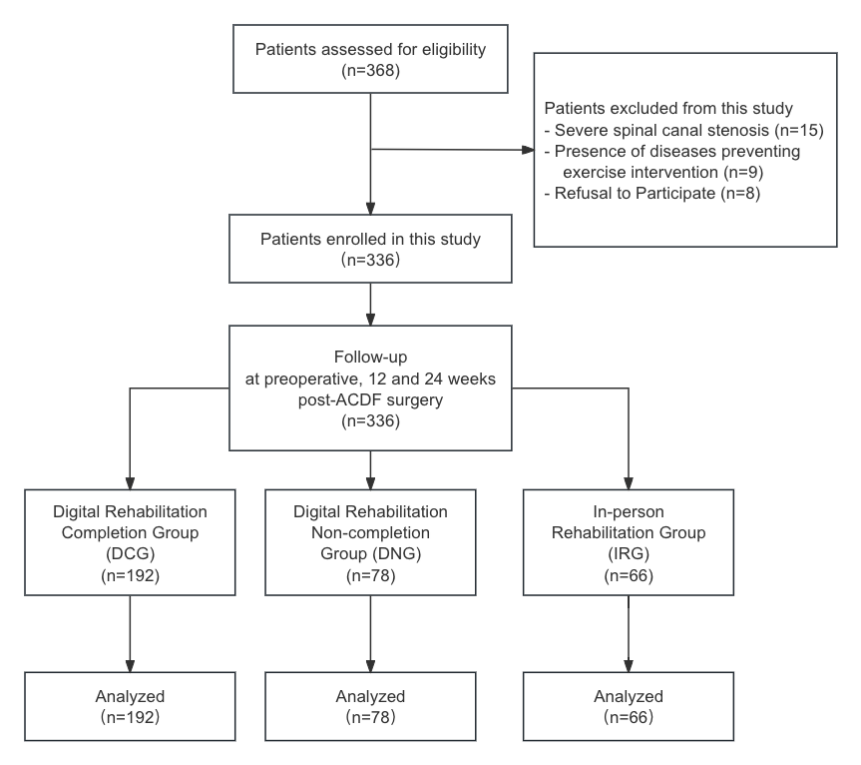
Experimental protocol.

Between January and December 2023, we screened 368 candidates for the study. Fifteen were excluded because of severe spinal-canal stenosis, 9 because of comorbid conditions that limited exercise, and 8 declined participation. The remaining 336 patients met all criteria and completed the baseline assessment (Figure 3). All were followed longitudinally and allocated to one of 3 cohorts according to actual rehabilitation uptake: 192 in the DCG, 78 in the DNG, and 66 in the IRG.

Baseline demographic and neurological profiles were well matched. Mean age was 54.3 (10.1) years in the DCG, 54.1 (9.7) years in the DNG, and 53.4 (9.5) years in the IRG. Women accounted for 50%, 52%, and 51% of each group, respectively. On preoperative neurological examination, the prevalence of unilateral radicular pain was similar (56%, 59%, and 57%, respectively), as was bilateral radicular pain (13%, 14%, and 15%). Rates of muscle weakness (57%, 55%, and 56%), sensory deficit (43%, 45%, and 44%), and a positive upper-limb traction test (80%, 77%, and 78%) also showed no meaningful differences. These findings confirm baseline comparability among the 3 groups (Table 1).

**Table 1.** Baseline patient characteristics for the 3 groups.

Description	DCG^a^ (n=192)	DNG^b^ (n=78)	IRG^c^ (n=66)
Age (years), mean (SD)	54.3 (10.1)	54.1 (9.7)	53.4 (9.5)
Patient sex, n (%)			
Male	96 (50)	37 (48)	32 (49)
Female	96 (50)	41 (52)	34 (51)
Neurological examination, n (%)			
Unilateral nerve root pain	107 (56)	46 (59)	38 (57)
Bilateral nerve root pain	25 (13)	11 (14)	10 (15)
Motor dysfunction	109 (57)	43 (55)	37 (56)
Sensory disturbance	82 (43)	35 (45)	29 (44)
Upper limb tension test	154 (80)	60 (77)	52 (78)

a DCG: digital rehabilitation completion group.

b DNG: digital rehabilitation noncompletion group.

c IRG: in-person rehabilitation group.

### Primary Outcomes

#### Pain Intensity (Visual Analog Scale [VAS])

At 12 weeks postoperation, mean VAS scores changed by −2.5 points in the DCG (95% CI −3.0 to −2.0) and by −2.9 points in the IRG (95% CI −3.8 to −2.0). In contrast, the DNG showed only a −0.8 point change (95% CI −1.6 to 0.0). By 24 weeks, the pain had decreased in both completion groups, −4.0 points in the DCG (95% CI −4.5 to −3.5) and −4.1 points in the IRG (95% CI −5.0 to −3.2). The difference between these 2 groups was not significant (*P*=.82), yet both outperformed the DNG, which changed by just −1.3 points (95% CI −2.1 to −0.5) (Table 2).

**Table 2.** Pain and functional outcomes.

Outcomes and groups		Baseline (0-wk)	*P* value^a^	12-wk, mean (SD)	Δ12-wk^b^ (95% CI)	*P* value^a^	24-wk, mean (SD)	Δ24-wk^c^ (95% CI)	*P* value^a^
VAS (0‐10)									
DCG^d^		6.1 (3.1)	DCG^d^/IRG^e^: .83	3.6 (1.3)	–2.5 (–3.0 to –2.0)	DCG^d^/IRG^e^: .56	2.1 (2.2)	–4.0 (–4.5 to –3.5)	DCG^d^/IRG^e^: .46
DNG^f^		6.2 (2.9)	DCG^d^/DNG^f^: .80	5.4 (2.1)	–0.8 (–1.6 to 0.0)	DCG^d^/DNG^f^: <.001	4.9 (2.2)	–1.3 (–2.1 to –0.5)	DCG^d^/DNG^f^: <.001
IRG^e^		6.0 (3.3)	IRG^e^/DNG^f^: .70	3.2 (1.9)	–2.8 (–3.7 to –1.9)	IRG^e^/DNG^f^: <.001	1.9 (1.8)	–4.1 (–5.0 to –3.2)	IRG^e^/DNG^f^: <.001
NDI (0‐100)									
DCG^d^		38.2 (22.1)	DCG^d^/IRG^e^: .95	31.4 (11.3)	–6.8 (–10.3 to –3.3)	DCG^d^/IRG^e^: .54	24.9 (18.2)	–13.3 (–17.4 to –9.2)	DCG^d^/IRG^e^: .80
DNG^f^		37.5 (22.3)	DCG^d^/DNG^f^: .81	35.3 (14.6)	–2.2 (–8.2 to 3.8)	DCG^d^/DNG^f^: .04	31.1 (19.1)	–6.4 (–13.0 to 0.2)	DCG^d^/DNG^f^: .02
IRG^e^		38.4 (21.8)	IRG^e^/DNG^f^: .80	30.3 (13.1)	–8.1 (–14.3 to –1.9)	IRG^e^/DNG^f^: .03	24.2 (19.3)	–14.2 (–21.3 to –7.1)	IRG^e^/DNG^f^: .03
SF-36 MCS									
DCG^d^		37.3 (23.8)	DCG^d^/IRG^e^: .84	46.3 (25.8)	9.0 (4-14)	DCG^d^/IRG^e^: <.001	58.7 (14.9)	+21.4 (17.4 to 25.4)	DCG^d^/IRG^e^: <.001
DNG^f^		36.8 (23.9)	DCG^d^/DNG^f^: .88	37.9 (23.4)	+1.1 (–6.4 to 8.6)	DCG^d^/DNG^f^: .001	44.6 (18.7)	+7.8 (–1.0 to 14.6)	DCG^d^/DNG^f^: <.001
IRG^e^		36.6 (24.2)	IRG^e^/DNG^f^: .96	41.7 (24.4)	+5.1 (–3.3 to 13.5)	IRG^e^/DNG^f^: .34	49.5 (16.4)	+12.9 (5.8 to 20.0)	IRG^e^/DNG^f^: .09

a *P* value: the significance level was set at *P*<.05

b Δ12wk: difference of change between baseline and the 12th week’s outcome

c Δ24wk: difference of change between baseline and the 24th week’s outcome

d DCG: digital rehabilitation completion group.

e IRG: in-person rehabilitation group.

f DNG: digital rehabilitation noncompletion group.

#### Neck-Specific Disability (Neck Disability Index [NDI])

At the 12-week assessment, NDI scores changed by −6.8 points in the DCG (95% CI −10.3 to −3.3) and by −8.1 points in the IRG (95% CI −14.3 to −1.9), whereas the DNG changed by only −2.2 points. By week 24, the changes were even greater, −13.3 points in the DCG (95% CI −17.4 to −9.2) and −14.2 points in the IRG (95% CI −21.3 to −7.1). These two groups did not differ significantly (*P*=.46), but both had greater changes than DNG’s −6.4 points (95% CI −13.0 to 0.2) (Table 2).

### Secondary Outcomes

#### Psychological Health (SF-36 Mental Component Summary [MCS])

At Week 12, the DCG showed a 9-point rise in MCS (95% CI 4.0‐14.0), whereas the IIRG improved by 5.1 points (95% CI –3.3 to 13.5). The DNG gained only 1.1 points (95% CI –6.4 to 8.6). By Week 24, the DCG’s benefit widened to 21.4 points (95% CI 17.4‐25.4), significantly surpassing the IRG’s 12.9-point increase (95% CI 5.8‐20.0; between-group *P*<.05) and the DNG’s 7.8-point change (95% CI –1.0 to 14.6; between-group *P*<.05) (Table 2).

### Cervical Range of Motion (ROM)

#### Sagittal Plane (Flexion–Extension)

At postoperative 0 week, sagittal plane ROM was similar across groups with no statistically significant differences. At 12 weeks, sagittal ROM improved from 78.7° to 94.4° in the DCG, from 77.3° to 83.4° in the DNG, and from 77.9° to 93.2° in the IRG. The corresponding gains were 15.7° (95% CI 14.1-17.3), 6.1° (95% CI 3.6-8.6), and 15.3° (95% CI 12.7-17.9), respectively. By Week 24, DCG and IRG further improved to 102.4° and 104.5°, while DNG reached 89.2°. Between-group differences were statistically significant for DCG versus DNG and IRG versus DNG at both time points (*P*<.001).

##### Coronal Plane (Lateral Bending)

At postoperative 0 weeks, coronal plane ROM was similar across groups with no statistically significant differences. Coronal ROM increased from 56.7° to 69.8° in DCG, 57.4° to 63.8° in DNG, and 55.9° to 68.2° in IRG at 12 weeks, with corresponding gains of 13.1°, 6.4°, and 12.3°. By Week 24, DCG reached 76.1°, IRG 77.5°, and DNG 67.2°, with continued between-group significance favoring DCG and IRG over DNG (*P*<.001) (Table 3).

**Table 3.** Cervical spine range-of-motion and neck-muscle endurance outcomes.

Outcomes and groups		Baseline	*P* value^a^	12-week, mean (SD)	Δ12-week^b^ (95% CI)	*P* value^a^	24-week, mean (SD)	Δ24-week^c^ (95% CI)	*P* value^a^
Sagittal ROM^d^ (°)									
DCG^e^		78.7 (6.1)	DCG^e^/IRG^f^: .32	94.4 (9.4)	15.7 (14.1-17.3)	DCG^e^/IRG^f^: .36	102.4 (8.4)	23.7 (22.3-25.3)	DCG^e^/IRG^f^: .11
DNG^g^		77.3 (6.4)	DCG^e^/DNG^g^: .10	83.4 (9.1)	6.1 (3.6-8.6)	DCG^e^/DNG^g^:<.001	89.2 (12.7)	11.9 (8.7-15.1)	DCG^e^/DNG^g^:<.001
IRG^f^		77.9 (5.5)	IRG^f^/DNG^g^: .54	93.2 (9.3)	15.3 (12.7-17.9)	IRG^f^/DNG^g^:<.001	104.5 (9.6)	26.6 (23.9-29.3)	IRG^f^/DNG^g^:<.001
Coronal ROM (°)									
DCG^e^		56.7 (6.5)	DCG^e^/IRG^f^: .38	69.8 (7.1)	13.1 (11.7-14.5)	DCG^e^/IRG^f^: .36	76.1 (4.3)	19.4 (18.3-20.5)	DCG^e^/IRG^f^: .21
DNG^g^		57.4 (7.1)	DCG^e^/DNG^g^: .45	63.8 (5.9)	6.4 (4.3-8.5)	DCG^e^/DNG^g^:<.001	67.2 (5.3)	9.8 (7.8-11.8)	DCG^e^/DNG^g^:<.001
IRG^f^		55.9 (6.3)	IRG^f^/DNG^g^: .18	68.2 (6.7)	12.3 (10.1-14.5)	IRG^f^/DNG^g^:<.001	77.5 (8.6)	21.6 (19-24.2)	IRG^f^/DNG^g^:<.001
Horizontal ROM (°)									
DCG^e^		108.5 (5.3)	DCG^e^/IRG^f^: .22	123.2 (9.5)	14.7 (13.2-16.2)	DCG^e^/IRG^f^: .10	128.2 (12.4)	19.7 (17.8-21.6)	DCG^e^/IRG^f^: .34
DNG^g^		109.7 (5.9)	DCG^e^/DNG^g^: .12	115.4 (12.2)	5.7 (2.7-8.7)	DCG^e^/DNG^g^:<.001	118.9 (15.3)	9.2 (5.5-12.9)	DCG^e^/DNG^g^:<.001
IRG^f^		109.4 (5.0)	IRG^f^/DNG^g^: .74	126.3 (14.1)	16.9 (13.3-20.5)	IRG^f^/DNG^g^:<.001	130.1 (14.3)	20.7 (17-24.4)	IRG^f^/DNG^g^:<.001
Ventral endurance									
DCG^e^		43.4 (9.3)	DCG^e^/IRG^f^: .52	48.1 (5.1)	4.7 (3.2-6.2)	DCG^e^/IRG^f^: .08	55.2(4.2)	11.8 (10.4-13.2)	DCG^e^/IRG^f^: .62
DNG^g^		42.4 (8.8)	DCG^e^/DNG^g^: .41	45.3 (5.0)	2.9 (0.6-5.2)	DCG^e^/DNG^g^:<.001	48.4 (3.8)	6.0 (3.9-8.1)	DCG^e^/DNG^g^:<.001
IRG^f^		44.2 (8.4)	IRG^f^/DNG^g^: .21	49.5 (5.8)	5.3 (2.8-7.8)	IRG^f^/DNG^g^:<.001	55.6 (6.1)	11.4 (8.9-13.9)	IRG^f^/DNG^g^:<.001
Dorsal endurance (s)									
DCG^e^		75.8 (3.9)	DCG^e^/IRG^f^: .48	85.2 (4.2)	9.4 (8.6-10.2)	DCG^e^/IRG^f^: .71	90.6 (6.3)	14.8 (13.7 to 15.9)	DCG^e^/IRG^f^: .75
DNG^g^		76.8 (6.3)	DCG^e^/DNG^g^: .20	79.4 (6.9)	2.6 (0.5-4.7)	DCG^e^/DNG^g^:<.001	80.4 (6.7)	3.6 (1.5 to 5.7)	DCG^e^/DNG^g^:<.001
IRG^f^		75.2 (6.6)	IRG^f^/DNG^g^: .14	84.8 (8.4)	9.6 (7-12.2)	IRG^f^/DNG^g^:<.001	90.3 (6.7)	15.1 (12.8-17.4)	IRG^f^/DNG^g^:<.001

a *P* value: the significance level was set at *P*<.05.

b Δ12 week: difference of change between baseline and the 12th week’s outcome.

c Δ24 week: difference of change between baseline and the 24th week’s outcome.

d ROM: range of motion.

e DCG: digital rehabilitation completion group.

f IRG: in-person rehabilitation group.

g DNG: digital rehabilitation noncompletion group.

##### Horizontal Plane (Axial Rotation)

At postoperative 0 week, horizontal plane ROM was similar across groups with no statistically significant differences. Horizontal ROM improved from 108.5° to 123.2° in DCG, 109.7° to 115.4° in DNG, and 109.4° to 126.3° in IRG by week 12, and further to 128.2° (DCG), 118.9° (DNG), and 130.1° (IRG) by Week 24. Between-group comparisons at both time points showed significant advantages for DCG and IRG over DNG (*P*<.001) (Table 3).

### Cervical Flexor and Extensor Endurance

#### Ventral Endurance (Deep Flexor Muscles)

At baseline, deep-flexor endurance was similar across groups: DCG 43.4 (9.3)s, DNG 42.4 (8.8)s, and IRG 44.2 (8.4)s (all *P*>.40). By Week 12, endurance improved to 48.1 (5.1)s in the DCG, 45.3 (5.0)s in the DNG, and 49.5 (5.8)s in the IRG. The increases from baseline were 4.7s (95% CI 3.2-6.2), 2.9 (95% CI 0.6-5.2), and 5.3 (95% CI 2.8-7.8), respectively. Between-group comparisons showed significant differences between DCG and DNG (*P*<.001) and IRG and DNG (*P*<.001), but not between DCG and IRG (*P*=.08).

At Week 24, endurance further increased to 55.2 (4.2)s in the DCG and 55.6 (6.1)s in the IRG, compared to 48.4 (3.8)s in the DNG. Improvements were 11.8s (95% CI 10.4- 13.2), 11.4s (95% CI 8.9-13.9), and 6.0s (95% CI 3.9-8.1), respectively, with sustained significance for DCG versus DNG and IRG versus DNG (both *P*<.001) (Table 3).

##### Dorsal Endurance (Extensor Muscles)

At baseline, extensor endurance was also comparable: DCG 75.8 (3.9)s, DNG 76.8 (6.3)s, IRG 75.2 (6.6)s (*P* >.10). At Week 12, the values increased to 85.2 (4.2)s (DCG), 79.4s (6.9)s (DNG), and 84.8s (8.4)s (IRG), with corresponding gains of 9.4, 2.6, and 9.6.

At Week 24, endurance rose to 90.6 (6.3)s (DCG), 80.4 (6.7)s (DNG), and 90.3 (6.7)s (IRG). The improvements from baseline were 14.8s (95% CI 13.7-15.9s), 3.6s (95% CI 1.5-5.7s), and 15.1s (95% CI 12.8-17.4s), respectively. DCG and IRG both significantly outperformed DNG (*P*<.001), while DCG and IRG remained comparable (*P*=.75) (Table 3).

### Patient Satisfaction (PSQ-R)

At Week 24, patient-reported satisfaction was high and comparable between the DCG and the IRG: DCG, 95.7 (6.1) (95% CI 94.8‐96.6) versus IRG, 95.9 (6.5) (95% CI 94.3‐97.5); *P*=.83. The DNG averaged 84.3 (7.9) (95% CI 82.5‐86.1). Satisfaction in the DNG was lower than in the DCG (*P*<.001) and IRG (*P*<.001), reaching statistical significance (Table 4).

**Table 4.** Patient satisfaction at 24 weeks (PSQ-R).

Group	24-week, mean (SD)	95% CI	*P* value^a^
DCG^b^ (n=192)	95.7 (6.1)	94.8‐96.6	DCG^b^/IRG^c^: .83
DNG^d^ (n=78)	84.3 (12.9)	82.5‐86.1	DCG^b^/DNG^d^: <.001
IRG^c^ (n=66)	95.9 (6.5)	94.3‐97.5	IRG^c^/DNG[Table-fn T4_FN4]: <.001

a *P* value: the significance level was set at *P*<.05.

b DCG: digital rehabilitation completion group.

c IRG: in-person rehabilitation group.

d DNG: digital rehabilitation noncompletion group.

## Discussion

### Principal Findings

We enrolled 336 patients who underwent ACDF and compared 3 rehabilitation strategies—a complete digital program, conventional in-person physiotherapy, and an incomplete digital program—at 12 and 24 weeks. Patients who finished the digital program (DCG) achieved pain (VAS) and neck-specific disability (NDI) reductions comparable to those in the in-person group (IRG) and superior to those in the digital group who did not complete the program (DNG). Changes in 3-plane cervical range of motion and in flexor/extensor muscle endurance were greater in both DCG and IRG than in the DNG. The DCG also reported the largest improvement in mental health (SF-36 MCS). Patient satisfaction remained high and similar between the DCG and IRG groups, whereas inadequate compliance in DNG substantially weakened treatment benefits.

### Digital Rehabilitation for Functional Recovery

This study validated the effectiveness of a digital rehabilitation program in facilitating functional recovery following ACDF surgery. At 24 weeks postsurgery, the DCG demonstrated superior improvements in cervical spine mobility across sagittal, coronal, and horizontal planes compared to the DNG, with a range of motion improved reaching 23.7 degrees, 19.4 degrees, and 19.7 degrees for DCG versus 11.9 degrees, 9.8 degrees, and 9.2 degrees for DNG, respectively. Moreover, significant improvements in neck muscle endurance were observed in the DCG, for anterior and posterior muscle groups at 11.8 seconds and 14.8 seconds, respectively, compared to 6 seconds and 3.6 seconds in the DNG. A notable reduction in the NDI of 13.3 for DCG and 6.4 for DNG was also documented, further showing the digital rehabilitation program’s effect in promoting cervical spine functional recovery.

This study highlighted the effectiveness of combining computer vision-guided exercises with sensor-based monitoring to deliver precise rehabilitation guidance postsurgery. The computer vision-guided approach allowed the physician to monitor whether patients completed the exercises correctly and detected when there was difficulty performing an exercise. By providing precise and real-time feedback via computer vision, the digital program allows the physician to monitor the patient remotely and to adjust the intensity of the exercise, ensuring that patients are engaged in suitable exercises, therefore improving the effectiveness of the rehabilitation process. The wearable sensor further monitored patient behavior to correct postures, which could facilitate recovery. These findings are consistent with previous studies that showed digital rehabilitation is comparable to traditional rehabilitation methods in terms of effectiveness [39]. This is further supported by DCG and IRG demonstrating similar outcomes, both superior to DNG, showcasing the potential of technology-enhanced programs to offer more customized and flexible patient management solutions [40].

### Cognitive Behavioral Therapy for Pain Management

Additionally, this study evaluated the impact of integrating CBT into a digital rehabilitation program to improve pain management and psychological health. The usage of a digital platform for CBT delivery allowed the patients to have continuous access to pain management content and strategies, irrespective of physical location or mobility restrictions. The digital program taught patients how to manage postsurgical pain and educated patients to focus on breathing exercises and guided exercises to decrease pain. The results showed that a notable reduction in the VAS pain score of 4.1 for DCG by the 24th week, compared to 1.3 for the DNG. This finding is consistent with previous studies that demonstrated CBT techniques, such as cognitive restructuring and coping strategy enhancement, significantly reduce pain intensity and distress associated with pain conditions [36]. This is consistent with the gate control theory of pain, which proposes that when non-nociceptive input predominates, such as tactile stimulation or other sensory modalities, the perception of pain is reduced [40]. Moreover, the emphasis on behavior modification strategies, encouraging active engagement in exercise and social activities, resonates with previous work that found lifestyle modifications play a vital role in mitigating the influence of pain on daily functioning and overall quality of life [41]. These findings demonstrate the importance of the cognitive-behavioral component of pain management.

### Improved Patient Satisfaction

This study’s findings showed that patients in the DCG reported high satisfaction scores comparable to those in the IRG, with scores of 95.7 and 95.9, respectively. In contrast, the DNG reported a significantly lower satisfaction score of 84.3 points. The higher patient satisfaction observed in both the DCG and IRG for cervical spine rehabilitation post-ACDF was potentially caused by several key factors. The digital rehabilitation program tailored the rehabilitation plans to individual needs and introduced the convenience of undergoing therapy at home, the ease of access, and personalization potentially improved patient satisfaction. This is supported by previous studies that highlighted digital health programs in promoting patient engagement and satisfaction by leveraging the convenience and flexibility of technology-based solutions [42].

Moreover, the interactive software used in the digital rehabilitation program made rehabilitation exercises more engaging and interactive, similar to gameplay. The incorporation of interactive experiences within the digital rehabilitation program introduces patient satisfaction in the rehabilitation process, a factor that not only aids in maintaining high levels of patient compliance but also enriches the psychological well-being of patients undergoing treatment [43,44]. This finding is similar to previous research, which asserts that the interactive components of digital health programs contribute to positive patient experiences and outcomes [45]. These elements together contribute to the overall increase in patient satisfaction.

### Mental Health

The SF-36 MCS results highlight the impact of the rehabilitation types on mental health post-ACDF cervical spine rehabilitation. Over the 24 weeks, the DCG and IRG saw significant mental health improvements, with scores rising of 21.4 and 12.9 points, respectively. The DNG showed a moderate improvement of 7.8 points. This is consistent with previous studies that showed digital rehabilitation platforms, particularly those incorporating cognitive-behavioral strategies, have a significant positive impact on mental health [46]. The improved mental health score could be due to the comprehensive approach of these systems. Particularly in digital rehabilitation, the inclusion of CBT provides essential psychological support, directly contributing to mental health improvements. Also, software increases the patient education frequency, which could have a more profound impact on the patient’s mental health improvement. This is in contrast with the standard of care patient education, which solely focused on postoperative wound care and joint function training, which did not address the mental well-being of the patient.

### Clinical Implications of Digital Rehabilitation

Our study shows that patients who completed the 12-week digital rehabilitation program experienced pain relief, functional gains, and improvements in three-plane cervical range of motion and muscle endurance that matched those achieved through conventional, clinic-based therapy at both 12 and 24 weeks after ACDF. In addition, the digital group reported better mental health scores and higher satisfaction, whereas participants with poor adherence realized only modest improvements. These findings reinforce earlier telerehabilitation research indicating that smartphone- and wearable-based interventions can equal in-person physiotherapy in clinical effectiveness while offering greater accessibility and adherence [5,47].

The platform we evaluated combines computer-vision motion analysis, inertial-sensor posture monitoring, and brief CBT modules within a single mobile app. This training-and-feedback loop provides patients with real-time corrective cues and allows licensed clinicians to remotely review performance data and provide feedback to the patients. The CBT component addresses postoperative mood and motivation, thereby supporting sustained engagement [48]. High satisfaction and completion rates in the DRG further attest to the platform’s ability to keep users actively involved [6,49].

From a health services perspective, digital rehabilitation offers several advantages. First, clinicians can monitor recovery more frequently without requiring patients to make repeated clinic visits, reducing travel time and associated costs. Second, continuous data captured by the software enables providers to identify early signs of nonadherence or postoperative psychological distress and intervene promptly. Finally, because its underlying technologies are disease-agnostic, a similar framework can be applied to other musculoskeletal diseases, providing a scalable template for modernizing postoperative rehabilitation care.

### Limitations

This investigation was conducted at a single, high-volume tertiary medical center, which may limit geographic and institutional generalizability; however, the center’s standardized surgical pathways and comprehensive follow-up enhance internal validity and provide a benchmark for future multicenter studies. Second, rehabilitation modality was determined by participant preference rather than random allocation, introducing the possibility of selection bias. Third, although patients who underwent multilevel fusion were excluded to ensure surgical homogeneity, the resulting cohort offers a clear view of single-level ACDF recovery and establishes a foundation for subsequent work in more complex cases. These considerations suggest that in the future, randomized and multicenter clinical trials can extend the present findings to broader patient populations and surgical scenarios.

### Conclusions

This investigation demonstrates that a 12-week digital rehabilitation program delivers postoperative pain relief and functional recovery comparable to conventional, clinic-based therapy following ACDF. Beyond matching these core outcomes, the digital pathway confers additional advantages in psychological well-being and maintains high levels of patient-reported satisfaction. By combining computer-vision–guided exercise, wearable sensor monitoring, and embedded cognitive-behavioral support, the platform enables effective remote rehabilitation for ACDF patients. High compliance translates directly into greater clinical benefit, underscoring engagement as a pivotal determinant of success in remote care models. These findings support digital rehabilitation as a clinically effective, resource-efficient, and patient-centered option for postoperative rehabilitation, with potential for broader applications in musculoskeletal care.

## Supplementary material

10.2196/60717 Multimedia Appendix 1 Digital rehabilitation program exercise protocol for patients after anterior cervical discectomy and fusion.

## Abbreviations

ACDF: anterior cervical discectomy and fusion
CBT: cognitive behavioral therapy
DCG: digital rehabilitation completion group
DNG: digital rehabilitation noncompletion group
HRS: healbone rehabilitation system
IMU: inertial measurement unit
IRG: in-person rehabilitation group
MCS: mental component summary
NDI: neck disability index
PSQ-R: Patient Satisfaction Questionnaire–Revised
ROM: range of motion
SF-36: 36-Item Short Form Survey
VAS: visual analog scale
