# Supplementary material for: Mobile-Based Digital Rehabilitation Program for Patients After Anterior Cervical Discectomy and Fusion: Prospective Cohort Study
Source: JMIR Rehabil Assist Technol. 2025 Dec 1;12:e60717. doi: 10.2196/60717 (PMC12671286; doi:10.2196/60717)
Supplement: Multimedia Appendix 1 [file rehab-v12-e60717-s001.docx]

Digital Rehabilitation Program for Patients After Anterior Cervical Discectomy and Fusion: A Prospective Cohort Study

Sen Liu^1^, Xin Chen^2^, Di Liu^4^, Ye Lin^3^, Yaping Chen^1^, Siyi Cai^1^

^1^Department of Orthopedics, Peking Union Medical College Hospital, Chinese Academy of Medical Sciences (PUMCH-CAMS), Beijing, China

^2^Beijing Jishuitan Hospital Affiliated to Capital Medical University, Beijing, China

^3^Pritzker School of Medicine, University of Chicago, Chicago, IL, United States

^4^Jiakangzhongzhi Technology Co., Beijing, China

**Corresponding Author:**

Siyi Cai, PhD

Department of Orthopedics

Peking Union Medical College Hospital
Chinese Academy of Medical Sciences and Peking Union Medical College

No.1 Shuaifuyuan, Dongcheng District Beijing,100730

China

Phone:86-010-69151188

Email: drcaisiyi@163.com

**Abstract**

**Background:** Anterior cervical discectomy and fusion (ACDF) is a common treatment for degenerative cervical spine disease, yet its frequent postoperative follow-up places substantial demands on both patients and healthcare systems. A digital program integrating computer-vision–guided exercise, wearable posture monitoring, and cognitive-behavioral therapy could provide remote monitoring and rehabilitation to alleviate this burden.

**Objective:** To evaluate the clinical effectiveness and compliance of a 12-week digital rehabilitation program after ACDF compared with conventional in-person therapy.

**Methods:** In this prospective cohort study, 336 postoperative patients self-selected digital (n = 270) or In-person Rehabilitation Group (n = 66) rehabilitation. Digital users were stratified into a Digital Rehabilitation Completion Group (DCG, n = 192) and a Digital Rehabilitation Noncompletion Group (DNG, n = 78). Outcomes—primarily pain (VAS) and disability (NDI), as well as SF-36 MCS, three-plane cervical ROM, muscle endurance, and patient satisfaction—were recorded at 0, 12, and 24 weeks postoperatively. All statistical analyses were performed using SPSS version 29.0. Results were reported as means, standard deviations, and 95% confidence intervals (CI).

**Results:** Both the DCG and IRG demonstrated significant pain relief and functional improvement at weeks 12 and 24, with no statistically significant differences between the two groups (P > 0.05). At week 12, the mean change in VAS from baseline was −2.5 (95% CI −3.0 to −2.0) in the DCG and −2.8 (95% CI −3.7 to −1.9) in the IRG; the corresponding changes in NDI were −6.8 (95% CI −10.3 to −3.3) and −8.1 (95% CI −14.3 to −1.9), respectively. At week 24, the DCG showed a mean VAS change of −4.0 (95% CI −4.5 to −3.5) and an NDI change of −13.3 (95% CI −17.4 to −9.2), whereas the IRG showed −4.1 (95% CI −5.0 to −3.2) and −14.2 (95% CI −21.3 to −7.1), respectively. In contrast, the DNG exhibited markedly smaller reductions in pain and disability at both time points (P < 0.05 vs DCG and IRG). At week 12, the mean DNG VAS change was −0.8 (95% CI −1.6 to 0.0) and the NDI change was −2.2 (95% CI −8.2 to 3.8). At week 24, the DNG changes were −1.3 (95% CI −2.1 to −0.5) for VAS and −6.4 (95% CI −13.0 to 0.2) for NDI.

**Conclusion:** The digital rehabilitation program delivered clear clinical benefits in pain reduction, functional recovery, and mental health after ACDF, achieving outcomes comparable to those of conventional in-person rehabilitation. Higher compliance was associated with greater treatment effects, demonstrating the importance of sustained engagement. Accordingly, digital rehabilitation represents a clinically effective and patient-centered option for postoperative care following ACDF.

**Trial Registration**

This study was approved by the Ethics Committee of Peking Union Medical College Hospital (approval No. I-25PJ0581).

**Keywords**

Anterior Cervical Discectomy and Fusion; Digital Rehabilitation; Computer Vision; Remote Rehabilitation; Wearable Sensors; Postoperative Rehabilitation

**Introduction**

**Background**

With the rapid global aging of the population, degenerative cervical spine disease has become a major public health challenge affecting older adults. Approximately 85% of individuals above 60 years old show radiographic evidence of cervical degeneration, and the number of people living with neck pain reached 203 million—an increase of 77.3% since 1990—placing neck pain among the leading contributors to musculoskeletal disability^[1]^. Also, the global age-standardized prevalence of neck pain is 27.0 per 1000 in 2019, causing substantial health-care expenditure and productivity loss^[2]^.

For degenerative cervical spine disease accompanied by spinal cord or nerve-root compression, anterior cervical discectomy and fusion (ACDF) remains the surgical gold standard^[3]^. However, shorter inpatient stays indicate that most functional recovery occurs after discharge, and traditional outpatient physiotherapy often lacks continuous monitoring, real-time feedback, and psychological support^[4]^. It is further revealed that postoperative patients and their caregivers widely seek support to close these post-discharge guidance gaps^[4]^.

Digital rehabilitation has the potential to address these shortcomings and provide remote, real-time, and individualized exercise guidance. In previous studies involving chronic low back pain, AI-assisted tele-rehabilitation significantly reduced pain and improved function within 4–8 weeks^[5,6]^, while systematic reviews confirmed that digital health interventions lessen neck and back pain and enhance quality of life^[7,8]^. Other studies further indicate that spine-surgery patients using digital platforms experience lower rates of postoperative emergency visits and readmissions, highlighting their potential in postoperative care^[9]^.

However, evidence for digital rehabilitation specifically designed for ACDF postoperative patients remains limited^[9]^. Thus, this study evaluates the effectiveness and safety of an AI-driven digital rehabilitation program in promoting functional recovery and improving compliance following ACDF, thereby providing evidence-based postoperative digital rehabilitation strategies.

**Objective**

This study aims to evaluate the clinical efficacy of a digital rehabilitation program that combines computer-vision–guided exercise coaching, real-time inertial-sensor monitoring, and cognitive behavioral interventions for patients recovering from ACDF. By systematically comparing neck-pain intensity, quality of life, and functional recovery before and after the intervention, we will assess the program’s effectiveness in relieving symptoms, enhancing quality of life, and promoting functional recovery.

**Methods**

**Study Design**

This single-center, prospective cohort study was carried out at Peking Union Medical College Hospital to compare a 12-week digital rehabilitation program with standard in-person physiotherapy after ACDF^[10-15]^. A total of 336 eligible patients were enrolled.

Postoperatively, participants selected their rehabilitation modality based on personal preference and logistical convenience, forming the Digital Rehabilitation Group and the In-person Rehabilitation Group (IRG). Within the Digital Rehabilitation Group, compliance criteria further divided patients into a Digital Rehabilitation Completion Group (DCG) and a Digital Rehabilitation Noncompletion Group (DNG).

All groups followed an identical evidence-based rehabilitation protocol. Digital groups (DCG and DNG) used a tele-rehabilitation application paired with inertial-measurement-unit (IMU) wearables, completing three 30-minute sessions per week. In-person Rehabilitation Group (IRG) attended one 45-minute, in-persion session with a physical therapist each week and performed the remaining exercises independently at home using a printed manual. Pain, functional status, health-related quality of life, and cervical range of motion were assessed at baseline (week 0), week 12, and week 24, whereas patient satisfaction was measured only at week 24.

**Inclusion and Exclusion Criteria**

All participants were recruited at Peking Union Medical College Hospital. Two licensed physicians enrolled postoperative ACDF candidates according to the inclusion and exclusion criteria. Each participant received detailed information about the study’s objectives, procedures, and potential risks. Enrollment proceeded only after written informed consent was obtained. The inclusion and exclusion criteria were as follows:

**Inclusion Criteria**

(1) Less than 70 years old.

(2) Signed informed consent. (3) Diagnosis of cervical pathology Grade III or IV.

(4) Suffering from cervical spine disease for longer than 2 months.

(5) ≤ 3 segments requiring cervical fusion.

**Exclusion Criteria**

(1) Severe spinal canal stenosis.

(2) Presence of diseases preventing exercise.

(3) History of cervical or other spinal surgeries.

(4) History of spinal infection or tumors.

**Blinding**

Because of the distinct modes of intervention, conventional double‐blinding was not feasible. To minimize bias, we implemented several safeguards. Follow-up assessments were conducted by clinicians blinded to group allocation. Group identifiers were replaced with coded labels during data analysis, and data analysis was completed without knowledge of group allocation.

**Intervention**

The digital and in-person groups followed an identical rehabilitation protocol, designed by licensed physicians and physical therapists with reference to clinical guidelines. The program included cervical range-of-motion exercises, cervical-muscle strengthening exercises, scapular-stability exercises, and functional exercises. Full details are provided in Appendix 1. All patients underwent follow-up assessments at baseline, week 12, and week 24.

**Digital Rehabilitation Group**
 Participants used a mobile rehabilitation application paired with wearable inertial-measurement-unit (IMU) sensors for training and monitoring. They were instructed to complete three 30-minute sessions per week for 12 weeks, totaling 36 sessions according to clinical protocols^[16-18]^. The app comprises three modules: (1) computer-vision exercise coaching, (2) cognitive behavioral therapy (CBT), and (3) posture monitoring. Figure 1 depicts the implementation of these modules.

**In-person Rehabilitation Group**
 Participants attended one 45-minute, therapist-supervised session per week and received a printed home-exercise booklet identical to the digital protocol for self-directed practice in between visits. Two licensed physical therapists delivering the sessions completed three training workshops (≥1 hour each) before the beginning of this study to ensure protocol fidelity.


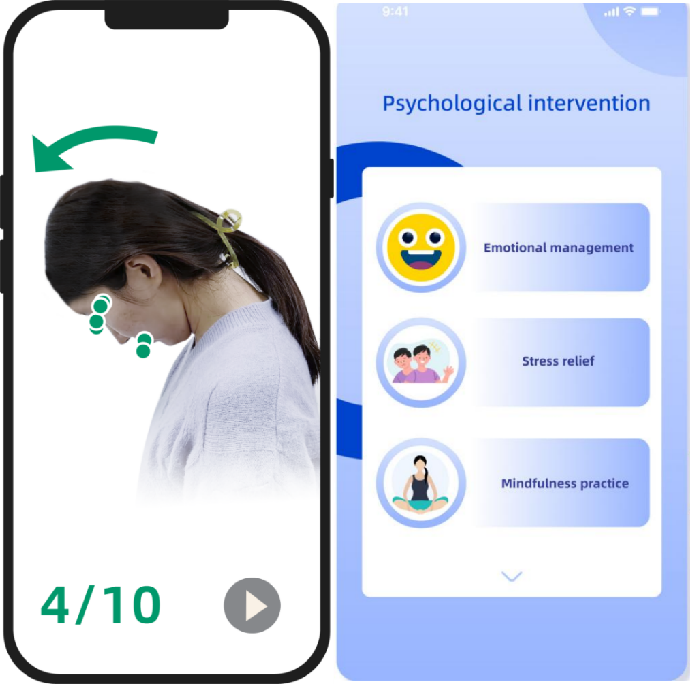


**Figure 1. Computer-vision–guided exercise and cognitive behavioral therapy modules.**

During each session, the front-facing cellphone camera works with a computer-vision-based software application to capture head-and-neck angles in real time for guided exercises. The cognitive behavioral therapy (CBT) module allows patients to access lessons and complete interactive homework assignments.


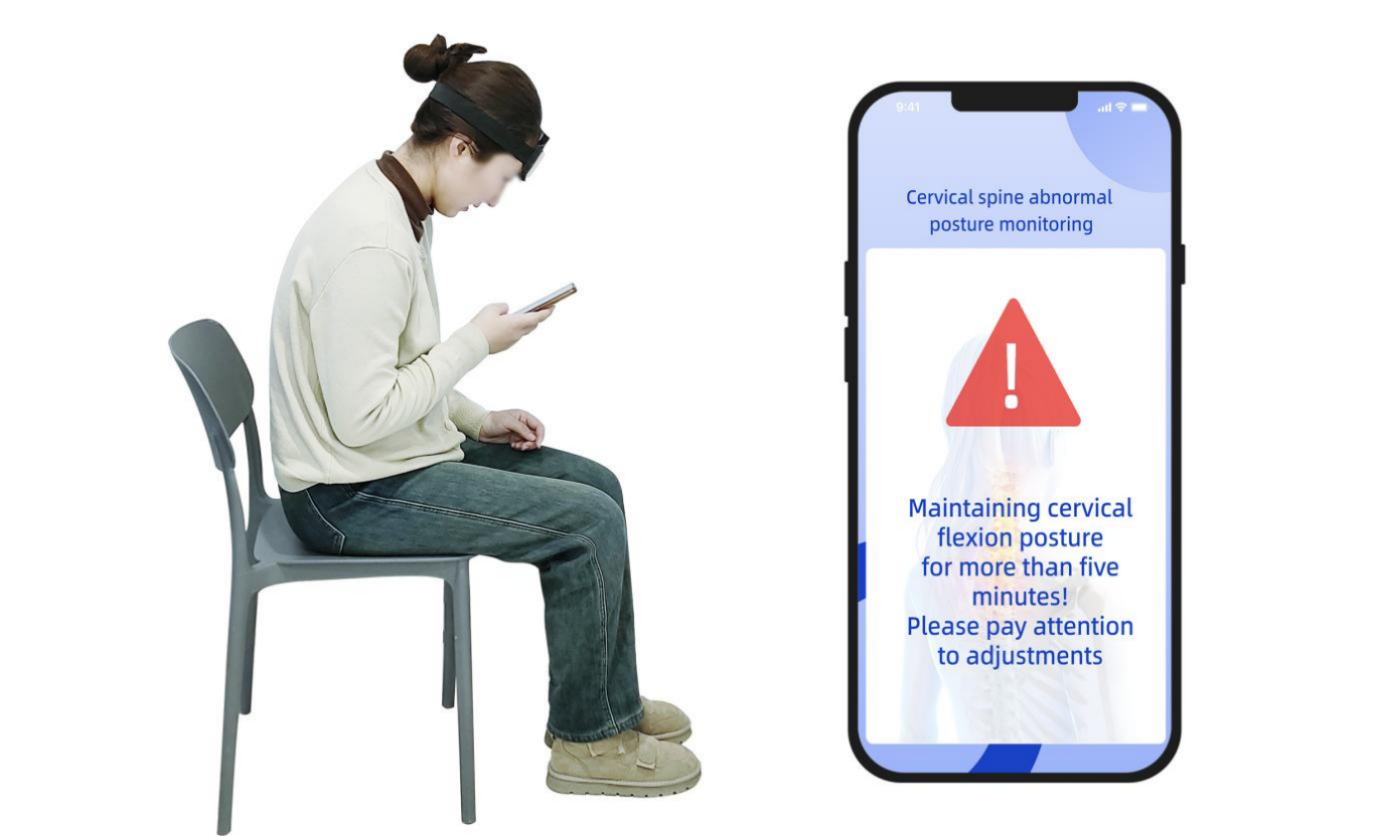


**Figure 2. Patient monitoring module and software module.**

The posture-monitoring module employs a 9-axis inertial measurement unit (IMU) paired with a notification alert system. Patients wear the IMU during daily activities; when abnormal cervical posture is detected, the system sends a warning message, encouraging proper postoperative posture.

**Computer-Vision Exercise Coaching**

Before discharge from the hospital, participants installed the training app on their smartphones and logged in with their registered accounts. Patients then followed the software application, which provided tutorials on how to complete each exercise. The cell phone’s front-facing camera captured cervical angles in real time, and the software provided verbal and auditory feedback to patients when they were completing their exercises in real time^[19-22]^. Each session lasted about 30 minutes, three times per week, for a total of 36 sessions over 12 weeks according to clinical guidelines^[23-27]^.

**Cognitive Behavioral Therapy (CBT)**

The digital rehabilitation app delivers eight micro-lessons designed to reduce postoperative chronic pain and psychological stress. Each lesson lasts 15–20 minutes and is completed once per week during the first eight weeks of training. Licensed therapists created the curriculum with reference to clinical protocols.The lessons progress through the following topics:

**Pain education:** Explaining that pain is not always proportional to tissue damage.

**Cognitive restructuring:** Recognizing and correcting negative thought patterns.

**Daily-routine planning:** Helping patients establish balanced activity and rest schedules.

**Sleep management:** Addressing postoperative insomnia or poor sleep quality.

**Stress regulation:** Mitigating anxiety, fear, and feelings of helplessness.

**Goal maintenance:** Reinforcing intrinsic motivation for long-term recovery.

After completing each lesson, patients complete an interactive homework assignment and write an electronic diary. Licensed therapists review submissions online each week and provide feedback within 24 hours, thereby fostering patients’ self-management skills.

**Posture Monitoring**

Patients in the digital rehabilitation group were instructed to wear a 9-axis inertial measurement unit (IMU) on the forehead for real-time tracking of cervical flexion and rotation. The device had to be worn for more than 4 hours per day during waking activities. If forward flexion exceeded or axial rotation surpassed a preset range, the application sent an alert. Each day, the software generated a posture-monitoring report and uploaded it to the cloud for therapist review. If no data were received for 48 hours, the system issued an automatic reminder to the patient, followed by a therapist phone check-in to improve engagement^[28-31]^.

**Completion Criteria**

Within the digital rehabilitation arm, patients were classified as either the Digital Rehabilitation Completion Group (DCG) or the Digital Rehabilitation Noncompletion Group (DNG) according to the following criteria:

**Exercise compliance:** All 36 scheduled sessions had to be completed. Each session required every prescribed exercise to be completed.

**CBT compliance:** All eight CBT micro-lessons had to be viewed, with the associated homework submitted.

**Posture-monitoring compliance:** The inertial measurement unit (IMU) had to be worn for ≥ 4 hours per day, on ≥ 70% of the total days for the 12 week digital program^[32-34]^.

Every DCG patient completed the full set of 36 sessions and met the CBT and posture-monitoring requirements. By contrast, most DNG participants discontinued the program early and did not meet completion criteria.

**Outcome Measures**

**Primary Outcomes**

Primary outcomes included pain intensity, assessed with a Visual Analogue Scale (VAS), and neck-specific disability, assessed with the Neck Disability Index (NDI). These measures are well validated in cervical‐spine disease populations^[35]^. We measured both outcomes at 0, 12, and 24 weeks postoperatively to capture the longitudinal course of pain relief and functional recovery.

**Secondary Outcomes**

**Psychological Health**

Psychological health was assessed with the Mental Component Summary (MCS) of the 36-Item Short Form Survey (SF-36)^[36]^. Higher MCS values indicate better mental well-being. Participants complete the SF-36 at 0, 12, and 24 weeks after surgery, allowing us to track the trajectory of postoperative psychological adjustment alongside physical recovery.

**Objective Kinematics**

Cervical mobility was quantified as active range of motion (ROM) in three anatomical planes—flexion–extension, lateral bending, and axial rotation—using a handheld digital inclinometer. After a standardized warm-up, each participant performs three maximal pain-free movements per plane; the mean of the three trials is recorded at 0, 12, and 24 weeks to document changes in kinematics over time.

**Muscle Endurance**

Cervical flexor and extensor endurance was measured with standardized supine and prone hold tests as described by Harris et al.^[37]^. For the flexor assessment, the participant was in supine position, lifted their heads upwards and maintained this posture until they felt fatigued. The duration of the test was measured in seconds. For the extensor assessment, positioned in a prone posture, participants suspended their necks in the air with weight attached (2 kg for males, 1 kg for females), and maintained the neck parallel to the ground until they felt fatigued. The assessments were performed three times, and the mean of the three trials is recorded at 0, 12, and 24 weeks, providing an objective measure of muscular performance during rehabilitation.

**Patient Experience**

Overall patient experience was evaluated once, at 24 weeks, using the Chinese Patient Satisfaction Questionnaire–Revised (PSQ-R)^[38]^. Each item is rated on a five-point Likert scale, with higher scores indicating greater satisfaction. Administering the PSQ-R after the full intervention period captures patients’ comprehensive impressions of care quality and rehabilitation effectiveness.

**Statistical Analysis**

All statistical analyses were performed using SPSS software (version 29.0; IBM Corp). Descriptive statistics for baseline characteristics were reported as mean (standard deviation, SD) for continuous variables or number (percentage) for categorical variables. Between-group comparisons for continuous variables were conducted using independent-sample two-tailed t-tests under the assumption of normality. Categorical variables were compared using chi-square tests. Primary outcomes (VAS and NDI) and secondary outcomes (SF-36 MCS, cervical ROM, muscle endurance) were assessed at baseline (0 week), 12 weeks, and 24 weeks postoperatively. Results were presented as means, SDs, and 95% CI. Pairwise group comparisons between the DCG, DNG, and IRG were conducted at each time point using independent-sample two-tailed t-tests. A P value less than 0.05 was considered statistically significant.

**Ethical Considerations**

This study was supported by CAMS Innovation Fund for Medical Sciences (CIFMS) [Grant No. 2023-I2M-C&T-B-002] and the National High-Level Hospital Clinical Research Funding [Grant No. 2022-PUMCH-A-123]. The protocol adhered to the principles of the Declaration of Helsinki and was reviewed and approved by the Ethics Committee of Peking Union Medical College Hospital, Chinese Academy of Medical Sciences (approval No. I-25PJ0581). Written informed consent was obtained from all participants prior to enrollment. All personal data were coded and de-identified to ensure participant privacy and data security. No financial or other compensation was offered to participants.

**Results**

**Participant Characteristics**

Between January and December 2023, we screened 368 candidates for the study. Fifteen were excluded because of severe spinal-canal stenosis, nine because of comorbid conditions that limited exercise, and eight declined participation. The remaining 336 patients met all criteria and completed the baseline assessment (Figure 3). All were followed longitudinally and allocated to one of three cohorts according to actual rehabilitation uptake: 192 in the Digital Rehabilitation Completion Group (DCG), 78 in the Digital Rehabilitation Noncompletion Group (DNG), and 66 in the In-person Rehabilitation Group (IRG).

Baseline demographic and neurological profiles were well matched. Mean age was 54.3 ± 10.1 years in the DCG, 54.1 ± 9.7 years in the DNG, and 53.4 ± 9.5 years in the IRG. Women accounted for 50%, 52%, and 51% of each group, respectively. On preoperative neurological examination, the prevalence of unilateral radicular pain was similar (56%, 59%, and 57%, respectively), as was bilateral radicular pain (13%, 14%, 15%). Rates of muscle weakness (57%, 55%, 56%), sensory deficit (43%, 45%, 44%), and a positive upper-limb traction test (80%, 77%, 78%) also showed no meaningful differences. These findings confirm baseline comparability among the three groups (Table 1).


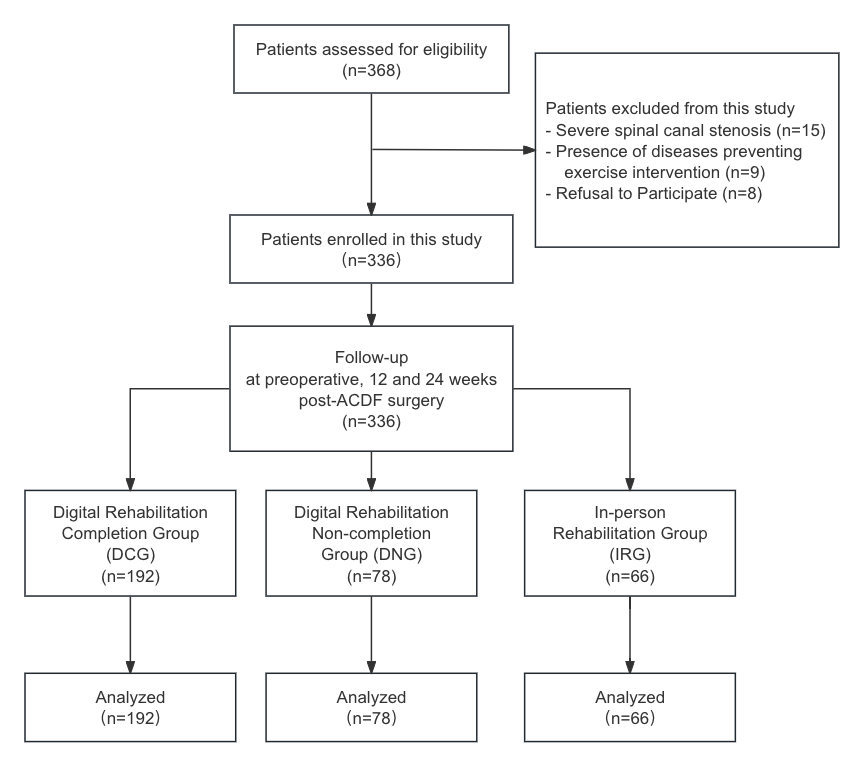


**Figure 3. Experimental Protocol.**

**Table 1. Baseline patient characteristic for the three groups.**

| **Description** | **DCG^a^**  **(n=192)** | **DNG^b^**  **(n=78)** | **IRG^c^**  **(n=66)** |
| --- | --- | --- | --- |
| **Age(years),mean(SD)** | 54.3(10.1) | 54.1(9.7) | 53.4(9.5) |
| **Patient sex, n (%)** |  |  |  |
| **Male** | 96(50%) | 37(48%) | 32(49%) |
| **Female** | 96(50%) | 41(52%) | 34(51%) |
| **Neurological Examination,n(%)** |  |  |  |
| **Unilateral Nerve Root Pain** | 107(56%) | 46(59%) | 38(57%) |
| **Bilateral Nerve Root Pain** | 25(13%) | 11(14%) | 10(15%) |
| **Motor Dysfunction** | 109(57%) | 43(55%) | 37(56%) |
| **Sensory Disturbance** | 82(43%) | 35(45%) | 29(44%) |
| **Upper Limb Tension Test** | 154(80%) | 60(77%) | 52(78%) |

^a^DCG: patients who completed digital rehabilitation program

^b^DNG: patients who started but did not complete digital rehabilitation program

^c^IRG: patients who opted for in-person rehabilitation

**Primary Outcomes**

**Pain Intensity (Visual Analogue Scale, VAS)**

At 12 weeks post-operation, mean VAS scores changed by −2.5 points in the Digital Rehabilitation Completion Group (DCG; 95% CI −3.0 to −2.0) and by −2.9 points in the In-person Rehabilitation Group (IRG; 95% CI −3.8 to −2.0). In contrast, the Digital Rehabilitation Noncompletion Group (DNG) showed only a −0.8 point change (95% CI −1.6 to 0.0). By 24 weeks, the pain had decreased in both completion groups, −4.0 points in the DCG (95% CI −4.5 to −3.5) and −4.1 points in the IRG (95% CI −5.0 to −3.2). The difference between these two groups was not significant (P = 0.82), yet both outperformed the DNG, which changed by just −1.3 points (95% CI −2.1 to −0.5) (Table 2).

**Neck-specific Disability (Neck Disability Index, NDI)**

At the 12-week assessment, NDI scores changed by −6.8 points in the DCG (95% CI −10.3 to −3.3) and by −8.1 points in the IRG (95% CI −14.3 to −1.9), whereas the DNG changed by only −2.2 points. By week 24, the changes were even greater, −13.3 points in the DCG (95% CI −17.4 to −9.2) and −14.2 points in the IRG (95% CI −21.3 to −7.1). These two groups did not differ significantly (P = 0.46), but both had greater changes than DNG’s −6.4 points (95% CI −13.0 to 0.2) (Table 2).

| **Outcome** | **Group** | **Baseline（0wk）** | **P value^g^** | **12 wk,mean (SD)** | **Δ12wk^e^(95% CI)** | **P value^g^** | **24 wk,mean (SD)** | **Δ24wk^f^(95% CI)** | **P value^g^** |
| --- | --- | --- | --- | --- | --- | --- | --- | --- | --- |
| **VAS (0–10)** | DCG^a^ | 6.1(3.1) | DCG^a^/IRG^c^: 0.83  DCG^a^/DNG^b^: 0.80 IRG^c^/DNG^b^: 0.70 | 3.6(1.3) | –2.5(-3.0 to -2.0) | DCG^a^/IRG^c^: 0.56  DCG^a^/DNG^b^: <0.01 IRG^c^/DNG^b^: <0.01 | 2.1(2.2) | –4.0(-4.5 to -3.5) | DCG^a^/IRG^c^: .46  DCG^a^/DNG^b^: <0.01 IRG^c^/DNG^b^: <0.01 |
|  | DNG^b^ | 6.2(2.9) |  | 5.4(2.1) | –0.8(-1.6 to 0.0) |  | 4.9(2.2) | –1.3(-2.1 to -0.5) |  |
|  | IRG^c^ | 6.0(3.3) |  | 3.2(1.9) | –2.8(-3.7 to -1.9) |  | 1.9(1.8) | –4.1(-5.0 to -3.2) |  |
| **NDI (0–100)** | DCG^a^ | 38.2(22.1) | DCG^a^/IRG^c^: 0.95  DCG^a^/DNG^b^: 0.81 IRG^c^/DNG^b^: 0.80 | 31.4(11.3) | –6.8(-10.3 to -3.3) | DCG^a^/IRG^c^: 0.54  DCG^a^/DNG^b^: 0.04 IRG^c^/DNG^b^: 0.03 | 24.9(18.2) | –13.3(-17.4 to -9.2) | DCG^a^/IRG^c^: 0.80  DCG^a^/DNG^b^: 0.02 IRG^c^/DNG^b^: 0.03 |
|  | DNG^b^ | 37.5(22.3) |  | 35.3(14.6) | –2.2(-8.2 to 3.8) |  | 31.1(19.1) | –6.4(-13.0 to 0.2) |  |
|  | IRG^c^ | 38.4(21.8) |  | 30.3(13.1) | –8.1(-14.3 to -1.9) |  | 24.2(19.3) | –14.2(-21.3 to -7.1) |  |
| **SF-36 MCS** | DCG^a^ | 37.3(23.8) | DCG^a^/IRG^c^: 0.84  DCG^a^/DNG^b^: 0.88 IRG^c^/DNG^b^: 0.96 | 46.3(25.8) | +9.0(4.0 to 14.0) | DCG^a^/IRG^c^: <0.01  DCG^a^/DNG^b^: 0.01 IRG^c^/DNG^b^: 0.34 | 58.7(14.9) | +21.4(17.4 to 25.4) | DCG^a^/IRG^c^: <0.01  DCG^a^/DNG^b^: <0.01 IRG^c^/DNG^b^: 0.09 |
|  | DNG^b^ | 36.8(23.9) |  | 37.9(23.4) | +1.1(-6.4 to 8.6) |  | 44.6(18.7) | +7.8(-1.0 to 14.6) |  |
|  | IRG^c^ | 36.6(24.2) |  | 41.7(24.4) | +5.1(-3.3 to 13.5) |  | 49.5(16.4) | +12.9(5.8 to 20.0) |  |

**Table 2 Pain and functional outcomes**

^a^DCG: patients who completed digital rehabilitation program

^b^DNG: patients who started but did not complete digital rehabilitation program

^c^IRG: patients who opted for in-person rehabilitation

^e^Δ12wk: difference of change between baseline and the 12th week’s outcome

^f^Δ24wk: difference of change between baseline and the 24th week’s outcome

^g^P value: the significance level was set at P < 0.05

**Secondary Outcomes**

**Psychological Health (SF-36 Mental Component Summary, MCS)**

At week 12, the Digital Rehabilitation Completion Group (DCG) showed a 9.0-point rise in MCS (95% CI 4.0–14.0), whereas the In-person Rehabilitation Group (IRG) improved by 5.1 points (95% CI –3.3 to 13.5). The Digital Rehabilitation Noncompletion Group (DNG) gained only 1.1 points (95% CI –6.4 to 8.6). By week 24, the DCG’s benefit widened to 21.4 points (95% CI 17.4–25.4), significantly surpassing the IRG’s 12.9-point increase (95% CI 5.8–20.0; between-group P < 0.05) and the DNG’s 7.8-point change (95% CI –1.0 to 14.6; between-group P < 0.05) (Table 2).

**Cervical Range of Motion (ROM)**

Baseline active ROM was similar across groups in all three planes—sagittal, coronal, and horizontal—with no statistically significant differences.

**Sagittal Plane (Flexion–Extension)**
 At 12 weeks, sagittal ROM improved from 78.7° to 94.4° in the DCG, from 77.3° to 83.4° in the DNG, and from 77.9° to 93.2° in the IRG. The corresponding gains were +15.7° (95% CI 14.1 to 17.3), +6.1° (95% CI 3.6 to 8.6), and +15.3° (95% CI 12.7 to 17.9), respectively. By week 24, DCG and IRG further improved to 102.4° and 104.5°, while DNG reached 89.2°. Between-group differences were statistically significant for DCG vs DNG and IRG vs DNG at both time points (P < 0.01).

**Coronal Plane (Lateral Bending)**
 Coronal ROM increased from 56.7° to 69.8° in DCG, 57.4° to 63.8° in DNG, and 55.9° to 68.2° in IRG at 12 weeks, with corresponding gains of +13.1°, +6.4°, and +12.3°. By week 24, DCG reached 76.1°, IRG 77.5°, and DNG 67.2°, with continued between-group significance favoring DCG and IRG over DNG (P < 0.01) (Table 3).

**Horizontal Plane (Axial Rotation)**
 Horizontal ROM improved from 108.5° to 123.2° in DCG, 109.7° to 115.4° in DNG, and 109.4° to 126.3° in IRG by week 12, and further to 128.2° (DCG), 118.9° (DNG), and 130.1° (IRG) by week 24. Between-group comparisons at both time points showed significant advantages for DCG and IRG over DNG (P < 0.01) (Table 3).

**Cervical Flexor and Extensor Endurance**

**Ventral Endurance (Deep Flexor Muscles)**
 At baseline, deep-flexor endurance was similar across groups: DCG 43.4 ± 9.3 s, DNG 42.4 ± 8.8 s, and IRG 44.2 ± 8.4 s (all P > .40). By week 12, endurance improved to 48.1 ± 5.1 s in the DCG, 45.3 ± 5.0 s in the DNG, and 49.5 ± 5.8 s in the IRG. The increases from baseline were +4.7 s (95% CI 3.2 to 6.2), +2.9 s (95% CI 0.6 to 5.2), and +5.3 s (95% CI 2.8 to 7.8), respectively. Between-group comparisons showed significant differences between DCG and DNG (P < 0.01) and IRG and DNG (P < 0.01), but not between DCG and IRG (P = 0.08).
 At week 24, endurance further increased to 55.2 ± 4.2 s in the DCG and 55.6 ± 6.1 s in the IRG, compared to 48.4 ± 3.8 s in the DNG. Improvements were +11.8 s (95% CI 10.4 to 13.2), +11.4 s (95% CI 8.9 to 13.9), and +6.0 s (95% CI 3.9 to 8.1), respectively, with sustained significance for DCG vs DNG and IRG vs DNG (both P < 0.01) (Table 3).

**Dorsal Endurance (Extensor Muscles)**
 At baseline, extensor endurance was also comparable: DCG 75.8 ± 3.9 s, DNG 76.8 ± 6.3 s, IRG 75.2 ± 6.6 s (P > .10). At week 12, the values increased to 85.2 ± 4.2 s (DCG), 79.4 ± 6.9 s (DNG), and 84.8 ± 8.4 s (IRG), with corresponding gains of +9.4 s, +2.6 s, and +9.6 s.
 At week 24, endurance rose to 90.6 ± 6.3 s (DCG), 80.4 ± 6.7 s (DNG), and 90.3 ± 6.7 s (IRG). The improvements from baseline were +14.8 s (95% CI 13.7 to 15.9), +3.6 s (95% CI 1.5 to 5.7), and +15.1 s (95% CI 12.8 to 17.4), respectively. DCG and IRG both significantly outperformed DNG (P < 0.01), while DCG and IRG remained comparable (P = 0.75) (Table 3).

| **Outcome** | **Group** | **Baseline** | **P value^g^** | **12 wk,mean (SD)** | **Δ12wk^e^(95% CI)** | **P value^g^** | **24 wk,mean (SD)** | **Δ24wk^f^(95% CI)** | **P value^g^** |
| --- | --- | --- | --- | --- | --- | --- | --- | --- | --- |
| **Sagittal ROM(°)** | DCG^a^ | 78.7(6.1) | DCG^a^/IRG^c^: .32  DCG^a^/DNG^b^: .10 IRG^c^/DNG^b^: .54 | 94.4(9.4) | +15.7(14.1 to 17.3) | DCG^a^/IRG^c^: .36  DCG^a^/DNG^b^: <.01 IRG^c^/DNG^b^: <.01 | 102.4(8.4) | +23.7(22.3 to 25.3) | DCG^a^/IRG^c^: .11  DCG^a^/DNG^b^: <.01 IRG^c^/DNG^b^: <.01 |
|  | DNG^b^ | 77.3(6.4) |  | 83.4(9.1) | +6.1(3.6 to 8.6) |  | 89.2(12.7) | +11.9(8.7 to 15.1) |  |
|  | IRG^c^ | 77.9(5.5) |  | 93.2(9.3) | +15.3(12.7 to 17.9) |  | 104.5(9.6) | +26.6(23.9 to 29.3) |  |
| **Coronal ROM (°)** | DCG^a^ | 56.7(6.5) | DCG^a^/IRG^c^: .38  DCG^a^/DNG^b^: .45 IRG^c^/DNG^b^: .18 | 69.8(7.1) | +13.1(11.7 to 14.5) | DCG^a^/IRG^c^: .36  DCG^a^/DNG^b^: <.01 IRG^c^/DNG^b^: <.01 | 76.1(4.3) | +19.4(18.3 to 20.5) | DCG^a^/IRG^c^: .21  DCG^a^/DNG^b^: <.01 IRG^c^/DNG^b^: <.01 |
|  | DNG^b^ | 57.4(7.1) |  | 63.8(5.9) | +6.4(4.3 to 8.5) |  | 67.2(5.3) | +9.8(7.8 to 11.8) |  |
|  | IRG^c^ | 55.9(6.3) |  | 68.2(6.7) | +12.3(10.1 to 14.5) |  | 77.5(8.6) | +21.6(19.0 to 24.2) |  |
| **Horizontal ROM(°)** | DCG^a^ | 108.5(5.3) | DCG^a^/IRG^c^: .22  DCG^a^/DNG^b^: .12 IRG^c^/DNG^b^: .74 | 123.2(9.5) | +14.7(13.2 to 16.2) | DCG^a^/IRG^c^: .10  DCG^a^/DNG^b^: <.01 IRG^c^/DNG^b^: <.01 | 128.2(12.4) | +19.7(17.8 to 21.6) | DCG^a^/IRG^c^: .34  DCG^a^/DNG^b^: <.01 IRG^c^/DNG^b^: <.01 |
|  | DNG^b^ | 109.7(5.9) |  | 115.4(12.2) | +5.7(2.7 to 8.7) |  | 118.9(15.3) | +9.2(5.5 to 12.9) |  |
|  | IRG^c^ | 109.4(5.0) |  | 126.3(14.1) | +16.9(13.3 to 20.5) |  | 130.1(14.3) | +20.7(17.0 to 24.4) |  |
| **Ventral endurance (s)** | DCG^a^ | 43.4(9.3) | DCG^a^/IRG^c^: .52  DCG^a^/DNG^b^: .41 IRG^c^/DNG^b^: .21 | 48.1(5.1) | +4.7(3.2 to 6.2) | DCG^a^/IRG^c^: .08  DCG^a^/DNG^b^: <.01 IRG^c^/DNG^b^: <.01 | 55.2(4.2) | +11.8(10.4 to 13.2) | DCG^a^/IRG^c^: .62  DCG^a^/DNG^b^: <.01 IRG^c^/DNG^b^: <.01 |
|  | DNG^b^ | 42.4(8.8) |  | 45.3(5.0) | +2.9(0.6 to 5.2) |  | 48.4(3.8) | +6.0 (3.9 to 8.1) |  |
|  | IRG^c^ | 44.2(8.4) |  | 49.5(5.8) | +5.3(2.8 to 7.8) |  | 55.6(6.1) | +11.4(8.9 to 13.9) |  |
| **Dorsal endurance (s)** | DCG^a^ | 75.8(3.9) | DCG^a^/IRG^c^: .48  DCG^a^/DNG^b^: .20 IRG^c^/DNG^b^: .14 | 85.2(4.2) | +9.4(8.6 to 10.2) | DCG^a^/IRG^c^: .71  DCG^a^/DNG^b^: <.01 IRG^c^/DNG^b^: <.01 | 90.6(6.3) | +14.8(13.7 to 15.9) | DCG^a^/IRG^c^: .75  DCG^a^/DNG^b^: <.01 IRG^c^/DNG^b^: <.01 |
|  | DNG^b^ | 76.8(6.3) |  | 79.4(6.9) | +2.6(0.5 to 4.7) |  | 80.4(6.7) | +3.6(1.5 to 5.7) |  |
|  | IRG^c^ | 75.2(6.6) |  | 84.8(8.4) | +9.6(7.0 to 12.2) |  | 90.3(6.7) | +15.1(12.8 to 17.4) |  |

**Table 3 Cervical spine range-of-motion (ROM) and neck-muscle endurance outcomes**

^a^DCG: patients who completed digital rehabilitation program

^b^DNG: patients who started but did not complete digital rehabilitation program

^c^IRG: patients who opted for in-person rehabilitation

^e^Δ12wk: difference of change between baseline and the 12th week’s outcome

^f^Δ24wk: difference of change between baseline and the 24th week’s outcome

^g^P value: the significance level was set at P < 0.05

**Patient Satisfaction (PSQ-R)**

At week 24, patient-reported satisfaction was high and comparable between the Digital Rehabilitation Completion Group (DCG) and the In-person Rehabilitation Group (IRG): DCG, 95.7 ± 6.1 (95% CI 94.8–96.6) versus IRG, 95.9 ± 6.5 (95% CI 94.3–97.5); P = 0.83. The Digital Rehabilitation Noncompletion Group (DNG) averaged 84.3 ± 7.9 (95% CI 82.5–86.1). Satisfaction in the DNG was lower than in the DCG (P < 0.01) and IRG (P < 0.01), reaching statistical significance (Table 4).

**Table 4 Patient satisfaction at 24 weeks (PSQ-R)**

| **Group** | **24 wk,mean (SD)** | **95% CI** | **P value^d^** |
| --- | --- | --- | --- |
| **DCG^a^(n = 192)** | 95.7(6.1) | 94.8–96.6 | DCG^a^/IRG^c^: .83  DCG^a^/DNG^b^: <.01 IRG^c^/DNG^b^: <.01 |
| **DNG^b^(n = 78)** | 84.3(12.9) | 82.5–86.1 |  |
| **IRG^c^(n = 66)** | 95.9(6.5) | 94.3–97.5 |  |

^a^DCG: patients who completed digital rehabilitation program

^b^DNG: patients who started but did not complete digital rehabilitation program

^c^IRG: patients who opted for in-person rehabilitation

^d^P value: the significance level was set at P < 0.05

**Discussion**

**Key Findings**

We enrolled 336 patients who underwent anterior cervical discectomy and fusion (ACDF) and compared three rehabilitation strategies— a complete digital program, conventional in-person physiotherapy, and an incomplete digital program— at 12 and 24 weeks. Patients who finished the digital program (DCG) achieved pain (VAS) and neck-specific disability (NDI) reductions comparable to those in the in-person group (IRG) and superior to those in the digital group who did not complete the program (DNG). Changes in three-plane cervical range of motion and in flexor/extensor muscle endurance were greater in both DCG and IRG than in the DNG. The DCG also reported the largest improvement in mental health (SF-36 MCS). Patient satisfaction remained high and similar between the DCG and IRG groups, whereas inadequate compliance in DNG substantially weakened treatment benefits.

**Digital Rehabilitation for Functional Recovery**

This study validated the effectiveness of a digital rehabilitation program in facilitating functional recovery following ACDF surgery. At 24 weeks post-surgery, the DCG demonstrated superior improvements in cervical spine mobility across sagittal, coronal, and horizontal planes compared to the DNG. With range of motion improved reaching 23.7 degrees, 19.4 degrees, and 19.7 degrees for DCG versus 11.9 degrees, 9.8 degrees, and 9.2 degrees for DNG, respectively. Moreover, significant improvements in neck muscle endurance were observed in the DCG, for anterior and posterior muscle groups at 11.8 seconds and 14.8 seconds, respectively, compared to 6 seconds and 3.6 seconds in the DNG. A notable reduction in the NDI of 13.3 for DCG and 6.4 for DNG was also documented, further showing the digital rehabilitation program's effect in promoting cervical spine functional recovery.

This study highlighted the effectiveness of combining computer vision guided exercises with sensor-based monitoring to deliver precise rehabilitation guidance post surgery. The computer vision guided approach allowed the physician to monitor whether patients completed the exercises correctly, and detected when there is difficulty performing an exercise. By providing precise and real-time feedback via computer vision, the digital program allows the physician to monitor the patient remotely and to adjust the intensity of the exercise, ensuring that patients are engaged in suitable exercises, therefore improving the effectiveness of the rehabilitation process. The wearable sensor further monitored patient behavior to correct postures which could facilitate recovery.These findings are consistent with previous studies that showed digital rehabilitation is comparable to traditional rehabilitation methods in terms of effectiveness^[39]^. This is further supported by DCG and IRG demonstrating similar outcomes, both superior to DNG, showcasing the potential of technology-enhanced programs to offer more customized and flexible patient management solutions^[40]^.

**Cognitive Behavioral Therapy for Pain Management**

Additionally, this study evaluated the impact of integrating cognitive behavioral therapy (CBT) into a digital rehabilitation program to improve pain management and psychological health. The utilization of a digital platform for CBT delivery allowed the patients to have continuous access to pain management content and strategies, irrespective of physical location or mobility restrictions. The digital program taught patients how to manage post-surgical pain and educated patients to focus on breathing exercises and guided exercises to decrease pain. The results showed that a notable reduction in the VAS pain score of 4.1 for DCG by the 24th week, compared to 1.3 for the DNG. This finding is consistent with previous studies that demonstrated CBT techniques, such as cognitive restructuring and coping strategy enhancement, significantly reduce pain intensity and distress associated with pain conditions^[36]^. This is consistent with gate control theory of pain which proposed that when non-nociceptive input predominates, such as tactile stimulation or other sensory modalities, the perception of pain is reduced^[40]^. Moreover, the emphasis on behavior modification strategies, encouraging active engagement in exercise and social activities, resonates with previous work that found lifestyle modifications play a vital role in mitigating the influence of pain on daily functioning and overall quality of life^[41]^. These findings demonstrate the importance of the cognitive behavioral component of pain management.

**Improved Patient Satisfaction**

This study's findings showed that patients in the DCG reported high satisfaction scores comparable to those in the IRG, with scores of 95.7 and 95.9 respectively. In contrast, the DNG reported a siginificantly lower satisfaction score of 84.3 points. The higher patient satisfaction observed in both the DCG and IRG for cervical spine rehabilitation post-ACDF was potentially caused by several key factors. The digital rehabilitation program tailored the rehabilitation plans to individual needs and introduced the convenience of undergoing therapy at home, the ease of access and personalization potentially improved patient satisfaction. This is supported by previous studies that highlighted digital health programs in promoting patient engagement and satisfaction by leveraging the convenience and flexibility of technology-based solutions^[42]^.

Moreover, the interactive software used in the digital rehabilitation program made rehabilitation exercises more engaging and interactive, similar to gameplay. The incorporation of interactive experiences within the digital rehabilitation program introduces patient satisfaction in the rehabilitation process, a factor that not only aids in maintaining high levels of patient compliance but also enriched the psychological well-being of patients undergoing treatment^[43-44]^. This finding is similar to previous research which asserts that the interactive components of digital health programs contribute to positive patient experiences and outcomes^[45]^. These elements together contribute to the overall increase in patient satisfaction.

**Mental Health**

The SF-36 MCS results highlight the impact of the rehabilitation types on mental health post-ACDF cervical spine rehabilitation. Over the 24 weeks, the DCG and IRG saw significant mental health improvements, with scores rising of 21.4 and 12.9 points, respectively. The DNG showed a moderate improvement of 7.8 points. This is consistent with previous studies that showed digital rehabilitation platforms, particularly those incorporating cognitive-behavioral strategies, have a significant positive impact on mental health^[46]^. The improved mental health scored could be due to the comprehensive approach of these systems. Particularly in digital rehabilitation, the inclusion of CBT provides essential psychological support, directly contributing to mental health improvements. Also software increases the patient education frequency, which could have a more profound impact on the patient’s mental health improvement. This is in contrast with standard of care patient education, which solely focused on postoperative wound care and joint function training, which did not address the mental well-being of the patient.

**Clinical Implications of Digital Rehabilitation**

Our study shows that patients who completed the 12-week digital rehabilitation program experienced pain relief, functional gains, and improvements in three-plane cervical range of motion and muscle endurance that matched those achieved through conventional, clinic-based therapy at both 12 and 24 weeks after ACDF. In addition, the digital group reported better mental-health scores and higher satisfaction, whereas participants with poor adherence realized only modest improvements. These findings reinforce earlier telerehabilitation research indicating that smartphone- and wearable-based interventions can equal in-person physiotherapy in clinical effectiveness while offering greater accessibility and adherence^[5, 47]^.

The platform we evaluated combines computer-vision motion analysis, inertial-sensor posture monitoring, and brief cognitive-behavioral therapy (CBT) modules within a single mobile application. This training-and-feedback loop provides patients with real-time corrective cues and allows licensed clinicians to remotely review performance data and provide feedback to the patients. The CBT component addresses postoperative mood and motivation, thereby supporting sustained engagement^[48]^. High satisfaction and completion rates in the DRG further attest to the platform’s ability to keep users actively involved^[6, 49]^.

From a health-services perspective, digital rehabilitation offers several advantages. First, clinicians can monitor recovery more frequently without requiring patients to make repeated clinic visits, reducing travel time and associated costs. Second, continuous data captured by the software enables providers to identify early signs of non-adherence or postoperative psychological distress and intervene promptly. Finally, because its underlying technologies are disease-agnostic, a similar framework can be applied to other musculoskeletal diseases, providing a scalable template for modernizing postoperative rehabilitation care.

**Limitations**

This investigation was conducted at a single, high-volume tertiary medical center, which may limit geographic and institutional generalizability; however, the center’s standardized surgical pathways and comprehensive follow-up enhance internal validity and provide a benchmark for future multicenter studies. Secondly, rehabilitation modality was determined by participant preference rather than random allocation, introducing the possibility of selection bias. Third, although patients who underwent multilevel fusion were excluded to ensure surgical homogeneity, the resulting cohort offers a clear view of single-level ACDF recovery and establishes a foundation for subsequent work in more complex cases. These considerations suggest that in the future, randomized and multicenter clinical trials can extend the present findings to broader patient populations and surgical scenarios.

**Conclusions**

This investigation demonstrates that a 12-week digital rehabilitation program delivers postoperative pain relief and functional recovery comparable to conventional, clinic-based therapy following ACDF. Beyond matching these core outcomes, the digital pathway confers additional advantages in psychological well-being and maintains high levels of patient-reported satisfaction. By combining computer-vision–guided exercise, wearable sensor monitoring, and embedded cognitive-behavioral support, the platform enables effective remote rehabilitation for ACDF patients. High compliance translated directly into greater clinical benefit, underscoring engagement as a pivotal determinant of success in remote care models. These findings support digital rehabilitation as a clinically effective, resource-efficient, and patient-centered option for postoperative rehabilitation, with potential for broader applications in musculoskeletal care.

**Ethics Approval**

The study protocol complied with the Declaration of Helsinki and was approved by the Ethics Committee of Peking Union Medical College Hospital, Chinese Academy of Medical Sciences (approval No. I-25PJ0581).

**Acknowledgements:** The authors would like to acknowledge Jiakangzhongzhi Technology Co. for providing access to this study population.

**Authors' Contributions:** SC was responsible for the initiation and planning of the project, as well as data collection, analysis, and interpretation. All contributors critically reviewed the manuscript for significant intellectual content and approved its final version for publication. SC acts as the custodian of the project.

**Data Availability:** Data available upon reasonable request.

**Conflicts of Interest:** None declared.

**Funding Support:** This research was funded by CAMS Innovation Fund for Medical Sciences（CIFMS）[Grant No. 2023-I2M-C&T-B-002] and the National High-Level Hospital Clinical Research Funding [Grant No. 2022-PUMCH-A-123].

**Ethics Approval:** This study complied with ethical standards and received approval from the Institutional Review Board (IRB) of Peking Union Medical College Hospital, Chinese Academy of Medical Sciences (approval No. I-25PJ0581)

**Provenance and Peer Review:** Not solicited; subject to external peer review.

**References**

[1]GBD 2021 Neck Pain Collaborators. Global, regional, and national burden of neck pain, 1990-2020, and projections to 2050: a systematic analysis of the Global Burden of Disease Study 2021. Lancet Rheumatol. 2024 Mar;6(3):e142-e155. doi: 10.1016/S2665-9913(23)00321-1. PMID: 38383088; PMCID: PMC10897950.

[2]Kazeminasab S, Nejadghaderi SA, Amiri P, Pourfathi H, Araj-Khodaei M, Sullman MJM, Kolahi AA, Safiri S. Neck pain: global epidemiology, trends and risk factors. BMC Musculoskelet Disord. 2022 Jan 3;23(1):26. doi: 10.1186/s12891-021-04957-4. PMID: 34980079; PMCID: PMC8725362.

[3]McKee C, Espey R, O'Halloran A, Curran A, Darwish N. A Retrospective Evaluation and Review of Radiographic Outcomes for Anterior Cervical Discectomy and Fusion (ACDF) Procedures: Northern Ireland's Experience. Cureus. 2023 May 11;15(5):e38864. doi: 10.7759/cureus.38864. PMID: 37205174; PMCID: PMC10188236.

[4]Ponder M, Ansah-Yeboah AA, Charalambous LT, Adil SM, Venkatraman V, Abd-El-Barr M, Haglund M, Grossi P, Yarbrough C, Dharmapurikar R, Gellad Z, Lad SP. A Smartphone App With a Digital Care Pathway for Patients Undergoing Spine Surgery: Development and Feasibility Study. JMIR Perioper Med. 2020 Oct 16;3(2):e21138. doi: 10.2196/21138. PMID: 33393924; PMCID: PMC7709850.

[5]Shi W, Zhang Y, Bian Y, Chen L, Yuan W, Zhang H, Feng Q, Zhang H, Liu D, Lin Y. The Physical and Psychological Effects of Telerehabilitation-Based Exercise for Patients With Nonspecific Low Back Pain: Prospective Randomized Controlled Trial. JMIR Mhealth Uhealth. 2024 Sep 6;12:e56580. doi: 10.2196/56580. PMID: 39240210; PMCID: PMC11395168.

[6]Xiao C, Zhao Y, Li G, Zhang Z, Liu S, Fan W, Hu J, Yao Q, Yang C, Zou J, Zeng Q, Huang G. Clinical Efficacy of Multimodal Exercise Telerehabilitation Based on AI for Chronic Nonspecific Low Back Pain: Randomized Controlled Trial. JMIR Mhealth Uhealth. 2025 May 22;13:e56176. doi: 10.2196/56176. Erratum in: JMIR Mhealth Uhealth. 2025 Jun 6;13:e78188. doi: 10.2196/78188. PMID: 40402551; PMCID: PMC12121543.

[7]Guo Q, Zhang L, Gui C, Chen G, Chen Y, Tan H, Su W, Zhang R, Gao Q. Virtual Reality Intervention for Patients With Neck Pain: Systematic Review and Meta-analysis of Randomized Controlled Trials. J Med Internet Res. 2023 Apr 3;25:e38256. doi: 10.2196/38256. PMID: 37010891; PMCID: PMC10131665.

[8]Valentijn PP, Tymchenko L, Jacobson T, Kromann J, Biermann CW, AlMoslemany MA, Arends RY. Digital Health Interventions for Musculoskeletal Pain Conditions: Systematic Review and Meta-analysis of Randomized Controlled Trials. J Med Internet Res. 2022 Sep 6;24(9):e37869. doi: 10.2196/37869. PMID: 36066943; PMCID: PMC9490534.

[9]Venkatraman V, Kirsch EP, Luo E, Kunte S, Ponder M, Gellad ZF, Liu B, Lee HJ, Jung SH, Haglund MM, Lad SP. Outcomes With a Mobile Digital Health Platform for Patients Undergoing Spine Surgery: Retrospective Analysis. JMIR Perioper Med. 2022 Oct 26;5(1):e38690. doi: 10.2196/38690. PMID: 36287589; PMCID: PMC9647464.

[10]Di Capua J, Somani S, Kim JS, Lee NJ, Kothari P, Phan K, Lugo-Fagundo N, Cho SK. Predictors for Patient Discharge Destination After Elective Anterior Cervical Discectomy and Fusion. Spine (Phila Pa 1976). 2017 Oct 15;42(20):1538-1544. doi: 10.1097/BRS.0000000000002140. PMID: 28252556.

[11]Katz AD, Mancini N, Karukonda T, Cote M, Moss IL. Comparative and Predictor Analysis of 30-day Readmission, Reoperation, and Morbidity in Patients Undergoing Multilevel ACDF Versus Single and Multilevel ACCF Using the ACS-NSQIP Dataset. Spine (Phila Pa 1976). 2019 Dec 1;44(23):E1379-E1387. doi: 10.1097/BRS.0000000000003167. PMID: 31348176.

[12]Guan L, Hai Y, Yang JC, Zhou LJ, Chen XL. Anterior cervical discectomy and fusion may be more effective than anterior cervical corpectomy and fusion for the treatment of cervical spondylotic myelopathy. BMC Musculoskelet Disord. 2015 Feb 13;16:29. doi: 10.1186/s12891-015-0490-9. PMID: 25881246; PMCID: PMC4344784.

[13]Astur N, Martins DE, Kanas M, Mendonça RGM, Creek AT, Lenza M, Wajchenberg M. Quality assessment of systematic reviews of surgical treatment of cervical spine degenerative diseases: an overview. Einstein (Sao Paulo). 2022 Apr 20;20:eAO6567. doi: 10.31744/einstein_journal/2022AO6567. PMID: 35476082; PMCID: PMC9000984.

[14]Ling J, Thirumavalavan J, Shin C, Lee TM, Marco RAW, Hirase T. Postoperative Rehabilitation to Improve Outcomes After Cervical Spine Fusion for Degenerative Cervical Spondylosis: A Systematic Review. Cureus. 2023 May 16;15(5):e39081. doi: 10.7759/cureus.39081. PMID: 37332472; PMCID: PMC10269395.

[15]Cerezci O, Basak AT. The Effects of Rehabilitation Following Anterior Cervical Microdiscectomy and Fusion Surgery. Turk Neurosurg. 2022;32(5):834-840. doi: 10.5137/1019-5149.JTN.36243-21.2. PMID: 35253156.

[16]Shim GY, Kim EH, Baek YJ, Chang WK, Kim BR, Oh JH, Lee JI, Hwang JH, Lim JY. A randomized controlled trial of postoperative rehabilitation using digital healthcare system after rotator cuff repair. NPJ Digit Med. 2023 May 23;6(1):95. doi: 10.1038/s41746-023-00842-7. PMID: 37221303; PMCID: PMC10204020.

[17]Wang P, Kong C, Teng Z, Zhang S, Cui P, Wang S, Zhao G, Lu S. Enhanced Recovery After Surgery (ERAS) Program for Anterior Cervical Discectomy and Fusion (ACDF) in Patients Over 60 Years Old. Clin Interv Aging. 2023 Sep 27;18:1619-1627. doi: 10.2147/CIA.S422418. PMID: 37790741; PMCID: PMC10543997.

[18]Debono B, Sabatier P, Boniface G, Bousquet P, Lescure JP, Garnaud V, Hamel O, Lonjon G. Implementation of enhanced recovery after surgery (ERAS) protocol for anterior cervical discectomy and fusion: a propensity score-matched analysis. Eur Spine J. 2021 Feb;30(2):560-567. doi: 10.1007/s00586-020-06445-0. Epub 2020 May 14. PMID: 32409887.

[19]Jia Y, Peng Z, Qin Y, Wang G. Surgical versus Nonsurgical Treatment for Adult Spinal Deformity: A Systematic Review and Meta-Analysis. World Neurosurg. 2022 Mar;159:1-11. doi: 10.1016/j.wneu.2021.12.017. Epub 2021 Dec 9. PMID: 34896664.

[20]Lao J, Yan M, Tian B, Jiang C, Luo C, Xie Z, Zhu Q, Bao Z, Zhong N, Tang X, Sun L, Wu G, Wang J, Peng H, Chu J, Duan C. Ultralow-Power Machine Vision with Self-Powered Sensor Reservoir. Adv Sci (Weinh). 2022 May;9(15):e2106092. doi: 10.1002/advs.202106092. Epub 2022 Mar 13. PMID: 35285175; PMCID: PMC9130913.

[21]Kim YJ, Nam HS, Lee WH, Seo HG, Leigh JH, Oh BM, Bang MS, Kim S. Vision-aided brain-machine interface training system for robotic arm control and clinical application on two patients with cervical spinal cord injury. Biomed Eng Online. 2019 Feb 11;18(1):14. doi: 10.1186/s12938-019-0633-6. PMID: 30744661; PMCID: PMC6371594.

[22]Mennel L, Symonowicz J, Wachter S, Polyushkin DK, Molina-Mendoza AJ, Mueller T. Ultrafast machine vision with 2D material neural network image sensors. Nature. 2020 Mar;579(7797):62-66. doi: 10.1038/s41586-020-2038-x. Epub 2020 Mar 4. PMID: 32132692.

[23]Karyotaki E, Efthimiou O, Miguel C, Bermpohl FMG, Furukawa TA, Cuijpers P; Individual Patient Data Meta-Analyses for Depression (IPDMA-DE) Collaboration; Riper H, Patel V, Mira A, Gemmil AW, Yeung AS, Lange A, Williams AD, Mackinnon A, Geraedts A, van Straten A, Meyer B, Björkelund C, Knaevelsrud C, Beevers CG, Botella C, Strunk DR, Mohr DC, Ebert DD, Kessler D, Richards D, Littlewood E, Forsell E, Feng F, Wang F, Andersson G, Hadjistavropoulos H, Christensen H, Ezawa ID, Choi I, Rosso IM, Klein JP, Shumake J, Garcia-Campayo J, Milgrom J, Smith J, Montero-Marin J, Newby JM, Bretón-Lopez J, Schneider J, Vernmark K, Bücker L, Sheeber LB, Warmerdam L, Farrer L, Heinrich M, Huibers MJH, Kivi M, Kraepelien M, Forand NR, Pugh N, Lindefors N, Lintvedt O, Zagorscak P, Carlbring P, Phillips R, Johansson R, Kessler RC, Brabyn S, Perini S, Rauch SL, Gilbody S, Moritz S, Berger T, Pop V, Kaldo V, Spek V, Forsell Y. Internet-Based Cognitive Behavioral Therapy for Depression: A Systematic Review and Individual Patient Data Network Meta-analysis. JAMA Psychiatry. 2021 Apr 1;78(4):361-371. doi: 10.1001/jamapsychiatry.2020.4364. Erratum in: JAMA Psychiatry. 2024 Mar 1;81(3):320. doi: 10.1001/jamapsychiatry.2023.5122. PMID: 33471111; PMCID: PMC8027916.

[24]Nagamitsu S, Kanie A, Sakashita K, Sakuta R, Okada A, Matsuura K, Ito M, Katayanagi A, Katayama T, Otani R, Kitajima T, Matsubara N, Inoue T, Tanaka C, Fujii C, Shigeyasu Y, Ishii R, Sakai S, Matsuoka M, Kakuma T, Yamashita Y, Horikoshi M. Adolescent Health Promotion Interventions Using Well-Care Visits and a Smartphone Cognitive Behavioral Therapy App: Randomized Controlled Trial. JMIR Mhealth Uhealth. 2022 May 23;10(5):e34154. doi: 10.2196/34154. PMID: 35604760; PMCID: PMC9171600.

[25]Nakao M, Shirotsuki K, Sugaya N. Cognitive-behavioral therapy for management of mental health and stress-related disorders: Recent advances in techniques and technologies. Biopsychosoc Med. 2021 Oct 3;15(1):16. doi: 10.1186/s13030-021-00219-w. PMID: 34602086; PMCID: PMC8489050.

[26]Rajabi Majd N, Broström A, Ulander M, Lin CY, Griffiths MD, Imani V, Ahorsu DK, Ohayon MM, Pakpour AH. Efficacy of a Theory-Based Cognitive Behavioral Technique App-Based Intervention for Patients With Insomnia: Randomized Controlled Trial. J Med Internet Res. 2020 Apr 1;22(4):e15841. doi: 10.2196/15841. PMID: 32234700; PMCID: PMC7160702.

[27]Sharma G, Schlosser L, Jones BDM, Blumberger DM, Gratzer D, Husain MO, Mulsant BH, Rappaport L, Stergiopoulos V, Husain MI. Brief App-Based Cognitive Behavioral Therapy for Anxiety Symptoms in Psychiatric Inpatients: Feasibility Randomized Controlled Trial. JMIR Form Res. 2022 Nov 2;6(11):e38460. doi: 10.2196/38460. PMID: 36322113; PMCID: PMC9669882.

[28]Wang Y, Zhou H, Yang Z, Samuel OW, Liu W, Cao Y, Li G. An intelligent wearable device for human's cervical vertebra posture monitoring. Annu Int Conf IEEE Eng Med Biol Soc. 2018 Jul;2018:3280-3283. doi: 10.1109/EMBC.2018.8512896. PMID: 30441092.

[29]Luo H, Jin T, Zhang Y, Tian B, Zhang Y, Wang D. A skin-integrated device for neck posture monitoring and correction. Microsyst Nanoeng. 2023 Nov 28;9:150. doi: 10.1038/s41378-023-00613-0. PMID: 38033991; PMCID: PMC10682425.

[30]Zhang Q, Li Y, Li Y, Yang X, Abbasi QH. Monitoring of neck activity for early warning of cervical spondylosis. Math Biosci Eng. 2022 Jan;19(1):792-811. doi: 10.3934/mbe.2022036. Epub 2021 Nov 22. PMID: 34903013.

[31]Lin PI, Chen TH, Chung HH, Su TM, Ma CC, Ou TC. Factors Associated with Postoperative Rehospitalization in Patients with Cervical Disc Herniation. Int J Environ Res Public Health. 2022 Feb 1;19(3):1687. doi: 10.3390/ijerph19031687. PMID: 35162713; PMCID: PMC8835259.

[32]Malliaras P, Cridland K, Hopmans R, Ashton S, Littlewood C, Page R, Harris I, Skouteris H, Haines T. Internet and Telerehabilitation-Delivered Management of Rotator Cuff-Related Shoulder Pain (INTEL Trial): Randomized Controlled Pilot and Feasibility Trial. JMIR Mhealth Uhealth. 2020 Nov 18;8(11):e24311. doi: 10.2196/24311. PMID: 33206059; PMCID: PMC7710452.

[33]Greenberg J, Popok PJ, Lin A, Kulich RJ, James P, Macklin EA, Millstein RA, Edwards RR, Vranceanu AM. A Mind-Body Physical Activity Program for Chronic Pain With or Without a Digital Monitoring Device: Proof-of-Concept Feasibility Randomized Controlled Trial. JMIR Form Res. 2020 Jun 8;4(6):e18703. doi: 10.2196/18703. PMID: 32348281; PMCID: PMC7308894.

[34]Mathiasen K, Riper H, Andersen TE, Roessler KK. Guided Internet-Based Cognitive Behavioral Therapy for Adult Depression and Anxiety in Routine Secondary Care: Observational Study. J Med Internet Res. 2018 Nov 28;20(11):e10927. doi: 10.2196/10927. PMID: 30487118; PMCID: PMC6291683.

[35]Parker SL, Godil SS, Shau DN, Mendenhall SK, McGirt MJ. Assessment of the minimum clinically important difference in pain, disability, and quality of life after anterior cervical discectomy and fusion: clinical article. J Neurosurg Spine. 2013 Feb;18(2):154-60. doi: 10.3171/2012.10.SPINE12312. Epub 2012 Nov 23. PMID: 23176164.

[36]Badhiwala JH, Witiw CD, Nassiri F, Akbar MA, Jaja B, Wilson JR, Fehlings MG. Minimum Clinically Important Difference in SF-36 Scores for Use in Degenerative Cervical Myelopathy. Spine (Phila Pa 1976). 2018 Nov 1;43(21):E1260-E1266. doi: 10.1097/BRS.0000000000002684. PMID: 29652783.

[37]Harris KD, Heer DM, Roy TC, Santos DM, Whitman JM, Wainner RS. Reliability of a measurement of neck flexor muscle endurance. Phys Ther. 2005 Dec;85(12):1349-55. PMID: 16305273.

[38]Niu Z, Huang L, He H, Mei S, Li L, Griffiths MD. The revised patient satisfaction questionnaire (PSQ-R): validity, reliability, equivalence, and network analysis among hospitalized patients in the Chinese population. BMC Health Serv Res. 2024 Oct 28;24(1):1289. doi: 10.1186/s12913-024-11788-1. PMID: 39468570; PMCID: PMC11520068.

[39]Zhang H, Wang J, Jiang Z, Deng T, Li K, Nie Y. Home-based tele-rehabilitation versus hospital-based outpatient rehabilitation for pain and function after initial total knee arthroplasty: A systematic review and meta-analysis. Medicine (Baltimore). 2023 Dec 22;102(51):e36764. doi: 10.1097/MD.0000000000036764. PMID: 38134064; PMCID: PMC10735162.

[40]Carl JR, Miller CB, Henry AL, Davis ML, Stott R, Smits JAJ, Emsley R, Gu J, Shin O, Otto MW, Craske MG, Saunders KEA, Goodwin GM, Espie CA. Efficacy of digital cognitive behavioral therapy for moderate-to-severe symptoms of generalized anxiety disorder: A randomized controlled trial. Depress Anxiety. 2020 Dec;37(12):1168-1178. doi: 10.1002/da.23079. Epub 2020 Jul 29. PMID: 32725848.

[41]Altug Z. Lifestyle Medicine for Chronic Lower Back Pain: An Evidence-Based Approach. Am J Lifestyle Med. 2021 Jul 21;15(4):425-433. doi: 10.1177/1559827620971547. PMID: 34366741; PMCID: PMC8299916.

[42]Gan DZQ, McGillivray L, Larsen ME, Christensen H, Torok M. Technology-supported strategies for promoting user engagement with digital mental health interventions: A systematic review. Digit Health. 2022 Jun 1;8:20552076221098268. doi: 10.1177/20552076221098268. PMID: 35677785; PMCID: PMC9168921.

[43]Chang WK, Lee JI, Hwang JH, Lim JY. Post-operative rehabilitation using a digital healthcare system in patients who had undergone rotator cuff repair: protocol for a single-center randomized controlled trial. Trials. 2022 Aug 17;23(1):667. doi: 10.1186/s13063-022-06648-4. PMID: 35978437; PMCID: PMC9386934.

[44]Zhang ZY, Tian L, He K, Xu L, Wang XQ, Huang L, Yi J, Liu ZL. Digital Rehabilitation Programs Improve Therapeutic Exercise compliance for Patients With Musculoskeletal Conditions: A Systematic Review With Meta-Analysis. J Orthop Sports Phys Ther. 2022 Nov;52(11):726-739. doi: 10.2519/jospt.2022.11384. Epub 2022 Aug 12. PMID: 35960507.

[45]Sardi L, Idri A, Fernández-Alemán JL. A systematic review of gamification in e-Health. J Biomed Inform. 2017 Jul;71:31-48. doi: 10.1016/j.jbi.2017.05.011. Epub 2017 May 20. PMID: 28536062.

[46]Rauschenberg C, Schick A, Hirjak D, Seidler A, Paetzold I, Apfelbacher C, Riedel-Heller SG, Reininghaus U. Evidence Synthesis of Digital Interventions to Mitigate the Negative Impact of the COVID-19 Pandemic on Public Mental Health: Rapid Meta-review. J Med Internet Res. 2021 Mar 10;23(3):e23365. doi: 10.2196/23365. PMID: 33606657; PMCID: PMC7951054.

[47]Bargeri S, Castellini G, Vitale JA, Guida S, Banfi G, Gianola S, Pennestrì F. Effectiveness of Telemedicine for Musculoskeletal Disorders: Umbrella Review. J Med Internet Res. 2024 Feb 2;26:e50090. doi: 10.2196/50090. PMID: 38306156; PMCID: PMC10873802.

[48]Davergne T, Meidinger P, Dechartres A, Gossec L. The Effectiveness of Digital Apps Providing Personalized Exercise Videos: Systematic Review With Meta-Analysis. J Med Internet Res. 2023 Jul 13;25:e45207. doi: 10.2196/45207. Erratum in: J Med Internet Res. 2023 Sep 21;25:e52522. doi: 10.2196/52522. PMID: 37440300; PMCID: PMC10375281.

[49]Vincent R, Charron M, Lafrance S, Cormier AA, Kairy D, Desmeules F. Investigating the Use of Telemedicine by Health Care Providers to Diagnose and Manage Patients With Musculoskeletal Disorders: Systematic Review and Meta-Analysis. J Med Internet Res. 2024 Sep 23;26:e52964. doi: 10.2196/52964. PMID: 39312765; PMCID: PMC11459102.

**Abbreviations**

**ACDF:** Anterior Cervical Discectomy and Fusion

**DCG:** Digital Rehabilitation Completion Group

**DNG:** Digital Rehabilitation Noncompletion Group

**IRG:** In-person Rehabiliation Group

**IMU:** Inertial Measurement Unit

**CBT:** Cognitive Behavioral Therapy

**NDI:** Neck Disability Index

**VAS:** Visual Analogue Scale

**ROM:** Range of Motion

**SF-36:** 36-Item Short Form Survey

**MCS:** Mental Component Summary

**PSQ-R**: Patient Satisfaction Questionnaire–Revised
